# *In vivo* and *Post-synthesis* Strategies to Enhance the Properties of PHB-Based Materials: A Review

**DOI:** 10.3389/fbioe.2020.619266

**Published:** 2021-01-14

**Authors:** Rosa Turco, Gabriella Santagata, Iolanda Corrado, Cinzia Pezzella, Martino Di Serio

**Affiliations:** ^1^Department of Chemical Sciences, University of Naples Federico II, Complesso Universitario di Monte Sant'Angelo, Naples, Italy; ^2^Institute for Polymers, Composites and Biomaterials, National Council of Research, Pozzuoli, Italy; ^3^Department of Agricultural Sciences, University of Naples Federico II, Portici, Italy

**Keywords:** polyhydroxybutyrate, plasticizer, reactive processing, biopolymer, bio-based network

## Abstract

The transition toward “green” alternatives to petroleum-based plastics is driven by the need for “drop-in” replacement materials able to combine characteristics of existing plastics with biodegradability and renewability features. Promising alternatives are the polyhydroxyalkanoates (PHAs), microbial biodegradable polyesters produced by a wide range of microorganisms as carbon, energy, and redox storage material, displaying properties very close to fossil-fuel-derived polyolefins. Among PHAs, polyhydroxybutyrate (PHB) is by far the most well-studied polymer. PHB is a thermoplastic polyester, with very narrow processability window, due to very low resistance to thermal degradation. Since the melting temperature of PHB is around 170–180°C, the processing temperature should be at least 180–190°C. The thermal degradation of PHB at these temperatures proceeds very quickly, causing a rapid decrease in its molecular weight. Moreover, due to its high crystallinity, PHB is stiff and brittle resulting in very poor mechanical properties with low extension at break, which limits its range of application. A further limit to the effective exploitation of these polymers is related to their production costs, which is mostly affected by the costs of the starting feedstocks. Since the first identification of PHB, researchers have faced these issues, and several strategies to improve the processability and reduce brittleness of this polymer have been developed. These approaches range from the *in vivo* synthesis of PHA copolymers, to the enhancement of *post-synthesis* PHB-based material performances, thus the addition of additives and plasticizers, acting on the crystallization process as well as on polymer glass transition temperature. In addition, reactive polymer blending with other bio-based polymers represents a versatile approach to modulate polymer properties while preserving its biodegradability. This review examines the state of the art of PHA processing, shedding light on the green and cost-effective tailored strategies aimed at modulating and optimizing polymer performances. Pioneering examples in this field will be examined, and prospects and challenges for their exploitation will be presented. Furthermore, since the establishment of a PHA-based industry passes through the designing of cost-competitive production processes, this review will inspect reported examples assessing this economic aspect, examining the most recent progresses toward process sustainability.

## Introduction

The exploitation of fossil resources to satisfy the current demand for plastic materials is a serious threat for the environment, with consequences in terms of global warming, human health risks, and ecosystem toxicity (Harding et al., 2007). The superior chemical and physical properties of petro-plastics, which are responsible for their wide applicability, turn out into very low degradation rate in the environment, determining their accumulation as serious pollutants. European policies in relation to waste management, emission reduction, and sustainable development strongly encourage the search for new green solutions to the plastic issue (Directive 2008/98/EC on waste).

Polyhydroxyalkanoates (PHAs) are biodegradable and naturally synthesized polyesters, accumulated by various microorganisms as carbon, energy, and redox storage material, in response to stressful/unbalanced growth conditions. The discovery of polyhydroxybutyrate (PHB) accumulation in *Bacillus megaterium* dates back to 1926 by Lemoigne. Since then, several steps ahead have been done in the field, from the identification of other hydroxyalkanoic acid monomers (1974), to the cloning and characterization of the genes involved in PHA biosynthesis (1988) (Choi et al., 2020). In 1995, the occurrence of more than 91 PHA monomers had been reported, with this number increasing to up to 150 (R)-hydroxyalkanoic acids at present (Muneer et al., 2020). Other important milestones in this field, including the characterization of the first copolymers and the description of the PHA biosynthetic pathways (1990s), up to the elucidation of the first crystal structure of the main PHA biosynthetic enzymes (2017) (Kim et al., 2017), have contributed to progresses in PHA production process as well as in modulation of polymer composition (Sudesh et al., 2000; Choi et al., 2020).

According to their monomer chain length, PHAs have been classified into three main categories: short chain length (scl)-PHA (C4 and C5), medium chain length (mcl)-PHA (C ≥6), and long chain length (lcl)-PHA (C >14). Since their discovery and characterization, PHAs and particularly PHB gained intensive attention from the scientific community, being the first example of bio-based and biodegradable polyesters synthesized *in vivo* by microorganisms for the intracellular storage. Hence, differently from the other bio-based polymers, PHAs are polymerized by several bacterial strains, and being natural polyesters, they are considered the most easily biodegradable polymers in aerobic (soil, compost, and marine) and anaerobic (sewage sludge, digesters, and landfills) environments, thanks to the biotic degradative action of several bacterial and fungal enzymes. Furthermore, PHA degradation products are easily assimilated into usable products for microbial growth. As a matter of fact, also PLA is a bio-based polymer produced through fermentation of lactic acid, but it is chemically polymerized and, most noteworthy, it is a compostable polymer, making it unsuitable to reduce the plastic waste pollution (Meereboer et al., 2020).

Among the PHAs, polyhydroxybutyrate (PHB), a scl-PHA, is by far the most well-studied PHA polymer, accumulated to up to 80% of cell dry weight by native as well as recombinant microorganisms (Aldor and Keasling, 2003). Being thermoplastic, it can develop bio-plastics by exploiting the common processing methodologies widely used for the oil-derived polymers and biodegradable polyesters, i.e., film casting and blowing, injection molding, extrusion, thermoforming, etc. (Raza et al., 2018). Due to its mechanical and barrier properties, which are similar to those of oil-based polymers such as polypropylene, this material can be proposed as an excellent candidate to substitute petroleum-derived plastics increasingly drawing the commercial attention. Indeed, PHB high melting temperature and high tensile strength are similar to that of polypropylene, whereas its gas barrier properties result in its being even better and more promising than those of polypropylene and polyethylene terephthalate; in light of the above, PHB deserves many attention for its great potential in several applications such as in food packaging. As an assessment, in Table 1, the main properties of PHB compared with that of PP are detailed (Masood, 2017; Markl, 2018). In particular, the molar mass of the polymers, representing the measure of the distribution of the individual molar masses around an average value, is reported. For the PHB, this value is measured to be very high and depends on the type of microorganism used and the conditions adopted for the fermentation process, the growth rate of the polymer, and the final purification procedures. This parameter is very important, since it significantly influences molecular, processing, and final properties of the polymer, in particular its mechanical performance (dos Santos et al., 2018).

**Table 1:** Range of typical properties of PHB (Bucci et al., 2005; Bugnicourt et al., 2014; dos Santos et al., 2017; Keskin et al., 2017; Rajan et al., 2017).

**Property**	**Value**	**PP**
Density, ρ (g/cm^3^)	1.23	0.095
Glass transition temperature, T_g_ (°C)	4–7	−10
Melting temperature, T_m_ (°C)	175–180	176
Crystallinity, Xcr (%)	50–90	50–70
Young's modulus, YM (GPa)	1–2	1.5–1.7
Tensile strength, TS (MPa)	15–40	38
Elongation at break, EB (%)	1–15	4
Water vapor transmission rate, WVTR (g·mm/m^2^·day MPa)	1–5	0.2–0.4
Oxygen transmission rate, OTR (cc·mm/m^2^·day)	2	67.7

In addition, PHB is biocompatible, and its degradation inside the body occurs slowly. For this reason, PHB can be used both as a polymer drug carrier in case of gradual and controlled releasing kinetics and as scaffold in the field of tissue engineering (Degli Esposti et al., 2019; Babos et al., 2020). Anyway, despite the comparable features with synthetic polymers, PHB exploitation is strongly limited due to its high stiffness, brittleness, and narrow processability window.

Although this phenomenon is not clearly elucidated, it is assumed that the interlamellar amorphous chains are progressively tightened up resulting in an increase in the amorphous rigid fraction (Crétois et al., 2016). This outcome could be explained by two plausible hypothesis. The first one is related to a secondary crystallization slowly occurring during the storage at room temperature: small crystallites not only can bridge crystalline lamellae but also freeze the remaining amorphous PHB chains, leading to the embrittlement of the material (Di Lorenzo and Righetti, 2013). The second assumption is based on the physical aging of the amorphous phase strictly linked to the non-equilibrium character of the glassy state. Actually, based on this theory, two types of amorphous phase coexist: a mobile amorphous fraction (MAF) far from the crystalline lamellae and a rigid amorphous fraction (RAF), which, due to the interaction with the crystals, evidences restricted mobility; in this last case, the boundary Tg associated is disturbed by the presence of crystals, and the physical aging process occurs below this Tg (Esposito et al., 2016).

Actually, both phenomena have strong consequences on the physical properties of PHB such as crystallinity, impact strength, Young's modulus, toughness, and elongation at break that undergo continuous worsening even to several days above its processing (dos Santos et al., 2017).

From the structural point of view, crystallinity is strongly related to its regular structure, which in turn depends on the type of synthesis adopted to obtain it. The isotactic PHB is characterized by the only presence of a chiral carbon in absolute configuration R (Michel and Billington, 2012) and is produced by bacterial fermentation (Vroman and Tighzert, 2009), while the syndiotactic PHB is synthesized starting from the monomers with R and S configuration (Barham et al., 1984). In this way, the fermentation route allows to obtain the highest crystallinity. Depending on the several approaches used to synthesize and process PHB, it is possible to affirm that they shoot in a high range between 40 and 80% (dos Santos et al., 2017).

In addition to its marked brittleness, with very low deformability, PHB has a very strong susceptibility to rapid thermal degradation, posing serious problems to processing using conventional technologies for thermoplastics (Erceg et al., 2005). Indeed, the polyester undergoes thermal degradation and depolymerization at temperatures close to its melting point at around 180°C, so that the acceptable residence time in the processing equipment is severely limited to only few minutes (Erceg et al., 2005).

**Figure 1:**
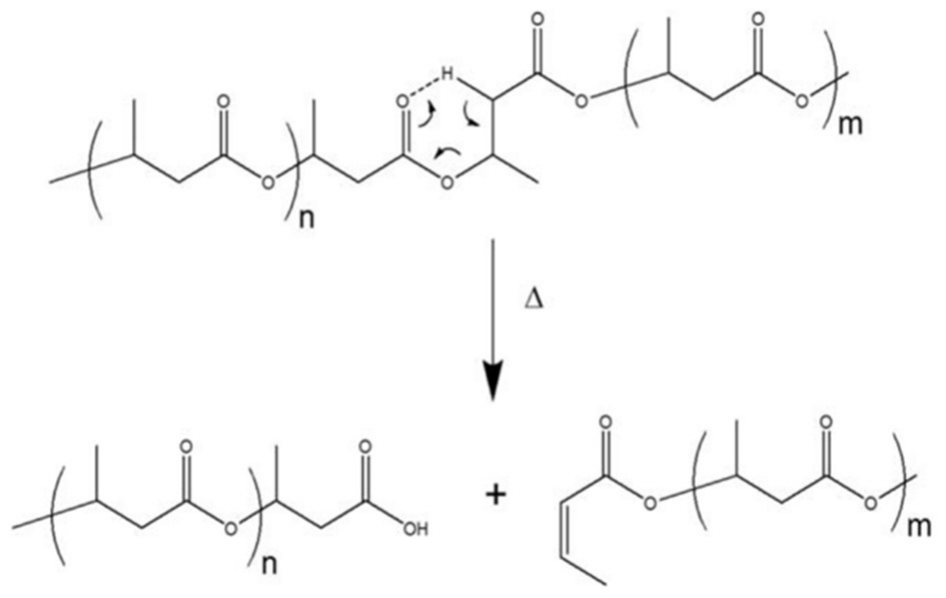
PHB β-elimination and degradation process.

The thermal degradation is believed to happen almost exclusively via a random chain cleavage mechanism. The degradative reaction, occurring with the elimination of cis β-CH and a six-membered ring transition (Gras et al., 1984; Doi et al., 1994), leads to a rapid decrease in molecular weight (Bugnicourt et al., 2014); however, some kinetically favorable cleavages occur near the ends of macromolecules (Erceg et al., 2005). Thus, several degraded products such as olefinic compounds, carboxylic acids, crotonic acid, and oligomers form (Figure 1).

Most of the bio-based polymers suffer from these shortcomings to such an extent that their diffusion as commodity materials has been confined to niche applications. As a result of the above flaws and to curb the previous drawbacks, several investigations reported in the literature have been performed aimed at producing PHB-based materials with improved properties.

**Figure 2:**
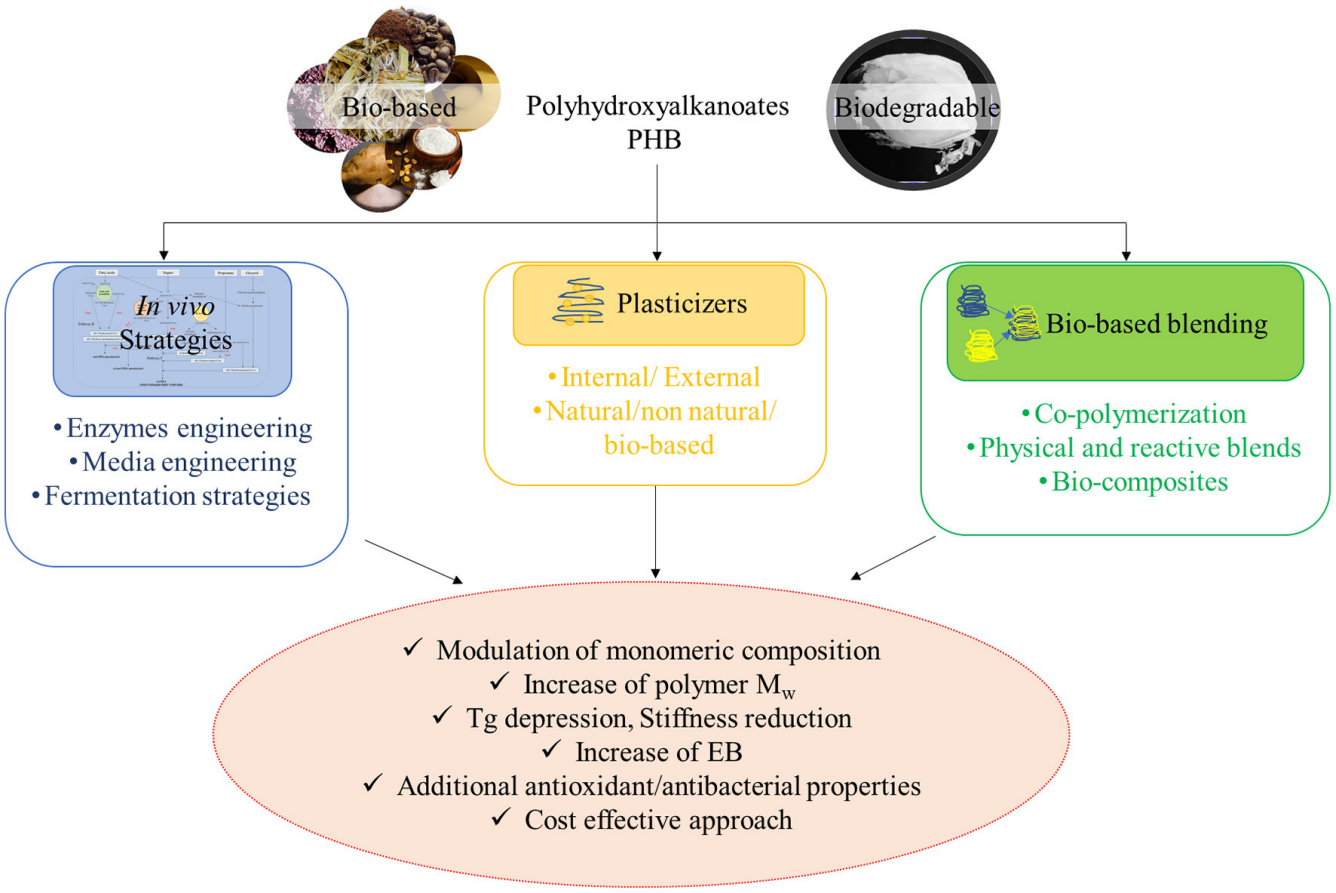
Green solutions for effective PHA processing and exploitation.

In this review, the most promising strategies finalized to obtain PHB-based materials with suitable and performing properties have been outlined (Figure 2). In particular, green and cost-effective approaches aimed to modulate and optimize the polymer technological performances have been discussed, as well as pioneering examples, prospects, and challenges for their effective exploitation have been detailed, too.

Specifically, in this review, two different approaches have been followed. The first one concerned the modification of the monomeric composition of PHA polymers; different kinds of PHAs have been *in vivo* biosynthesized by incorporating additional units into the PHB backbone, thus providing the possibility to fine tune the polymer properties: a general decrease in the glass transition and melting temperatures are observed with a consequent decreasing material brittleness and broadening processing window. In addition, *in vivo* biosynthetic methodologies aimed to modulate polymer molecular weight and cost competitiveness of the overall process have been also reviewed.

The second overture concerned the fine tuning of the properties of *post-synthesis* PHB-based polymers. To this aim, several studies related to PHB processing with compatible organic or inorganic materials, in order to obtain polymer physical blends, chemical reactive modifications, and bionanocomposites, have been recently reported, and a short account of them will be detailed in the following sections, together with the description of the main chemical–physical and mechanical properties. In particular, since the toughness and processability of PHB can be improved by incorporating natural additives, like plasticizers, a closer analysis on PHB, their main function, and of course, their most suitable plasticizers will be provided.

In this work, the literature data of the past 20 years, focusing specifically on the past decade, has been reviewed.

## *In vivo* Strategies to Modulate PHB Properties: Synthesis and Applications

### Copolymer Synthesis

The synthesis of copolymers of various molecular weights, compositions, and architectures has led to the ability to broaden the knowledge and the exploitation of new materials with unique properties. In addition to the control of the stereochemical microstructure, the copolymerization represents a way to opportunely modulate the physical properties of the polymeric materials. By varying the composition, monomer sequencing, and molecular weight, the polymer microstructure can be tailored for specific applications, mostly if a proper balance between the mechanical and thermal properties, together with degradation rate, is needed.

The introduction of other monomeric units in the PHB backbone has been reported to change polymer properties in favor of a reduced stiffness, higher elongation to break, and lower melting point.

**Figure 3:**
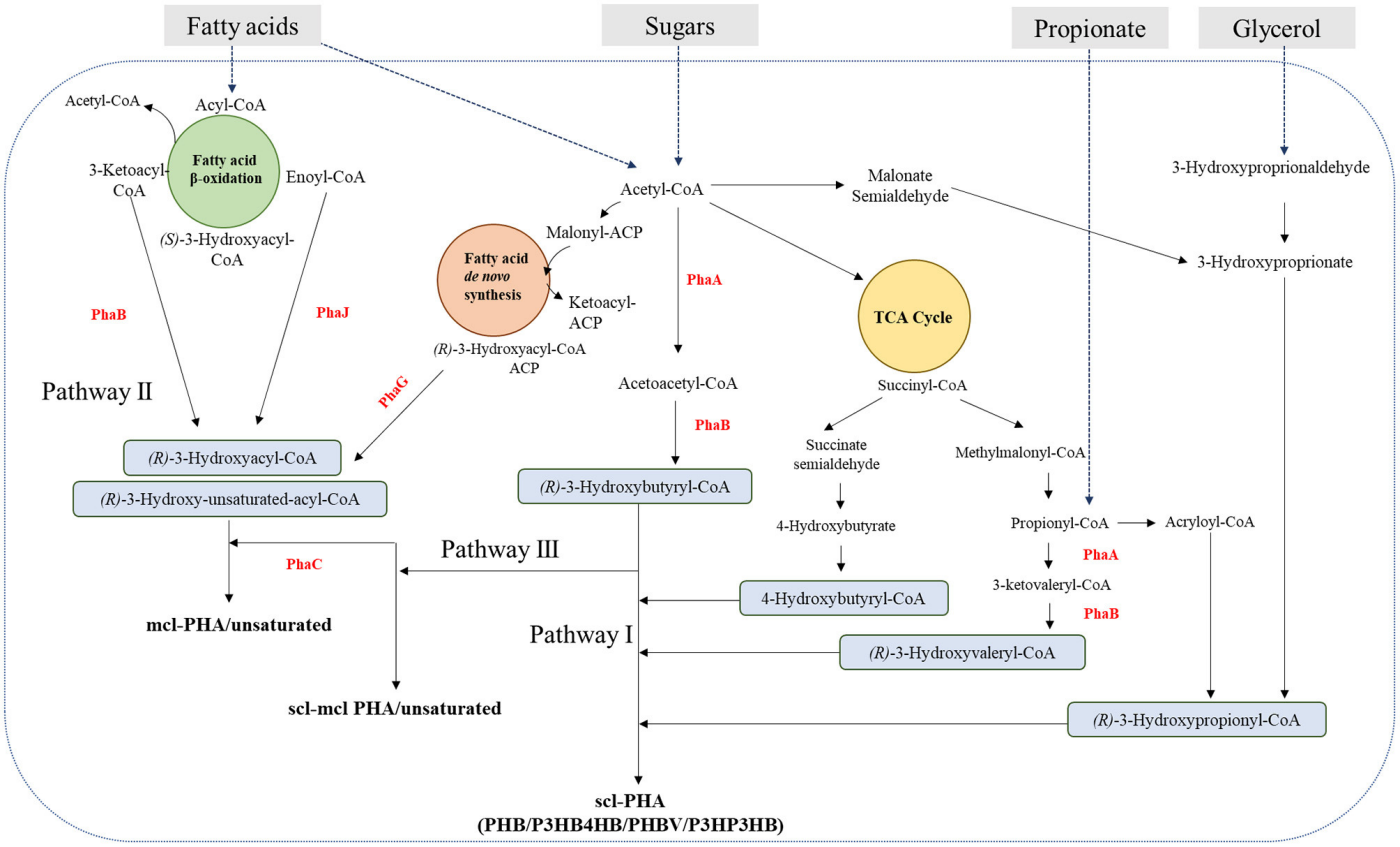
Biochemical pathways for PHAs production from different carbon sources. mcl, medium-chain-length; scl, short chain length; PhaB, Acetoacetyl-CoA reductase; PhaJ, Enoyl-CoA hydratase; PhaG, 3-hydroxyacyl-acyl carrier protein-coenzyme A transferase; PhaA, Acetyl-CoA acetyltranferase (β-ketothiolase); PhaC, PHA synthase; PHB, polyhydroxybutyrate; P3HB4HB, poly(3-hydroxybutyrate-*co*-4-hydroxybutyrate); PHBV, poly(3-hydroxybutyrate-*co*-hydroxyvalerate); P3HP3HB, poly(3-hydroxypropionate-*co*-3-hydroxybutyrate).

Copolymerization is obtained by the activation of different pathways for PHA biosynthesis in the microbial cell. Depending on the available carbon source, three main pathways regulate PHA biosynthesis *in vivo* (Figure 3), although up to 13 different routes allowing channeling specific precursors into PHA have been described (Tan et al., 2014b). If sugars are supplied, pathways I and III can be followed, yielding short chain length PHA (scl-PHA) and medium chain length PHA (mcl-PHA), respectively. These carbon sources are unrelated since their structure is different from that of PHA. If related carbon sources, such as fatty acids, are supplied, pathway II is followed, yielding mcl-PHA. Both pathways II and III can also produce PHA copolymers (Verlinden et al., 2007). It follows that the polymer composition is strictly related to the supplied C-source and to the activated pathway within the cell.

*In vivo* approaches for tailored copolymer synthesis are based on the use of both pure cultures and mixed microbial cultures (MMCs), in which the supplying of precursor compounds, structurally related to the monomer of interest, determines the activation of the metabolic pathway/s required for its synthesis. In the case of MMC, a raw complex organic substrate is first fermented to obtain volatile fatty acids (VFAs) by anaerobic acidogenic fermentation; then, the VFA-rich stream is used to select for PHA-producing microorganisms against the non-producing ones. Finally, the enriched microbial population is fed with the VFA under optimal conditions to maximize PHA production (Mannina et al., 2020). The type of starting feedstock, the operating conditions for VFA production, and their relative proportions, as well as the applied enrichment strategies directly affect the yield, productivity, and monomeric composition of the produced polymer (Kourmentza et al., 2017). Being based on natural principles of selection and competition among the microorganisms, MMC-based processes display an economic advantage since they are carried in unsterile conditions. Moreover, they allow to broaden the choice of possible feedstocks as the microbial consortium naturally adapts itself to the provided C-source (Mannina et al., 2020).

On the other hand, the use of pure cultures, although requiring sterile and thus costly conditions, is widely applied for PHA production (Anjum et al., 2016; Kourmentza et al., 2017). Both naturally occurring microorganisms as well as engineered strains have been used for copolymer synthesis, taking advantage of their heterogeneous metabolic pathways or finely manipulating them. Protein engineering, applied to the key enzymes of the biosynthetic pathway (Figure 3), also allowed to tailor their substrate specificity, favoring the incorporation of monomer of interests and, more recently, also of non-natural ones (Choi et al., 2020).

This section will cover the most significant examples in the field of copolymer synthesis. The mechanical and thermal properties of the most relevant examples of PHA copolymers have been collected in Table 2.

**Table 2:** Main properties of PHA copolymers.

**Copolymer**	**Strain, bioprocess**	**Properties**		**References**
		**Thermal**	**Mechanical**	
P(3HB-*co*-10.2 mol% 3HV)	*Ralstonia eutropha*, alternate feeding with glucose or propionate	M*_*w*_* (g mol^−1^) 6.7 × 10^−5^ T*_*m*_* (°C) 169 T*_*g*_* (°C) −1 T*_*c*_* (°C) 75	TS (MPa) 29.0 EB (%) 17.4 YM (MPa) 472	Madden et al., 1998
P(3HB-*co*-18.1 mol% 3HV)		M*_*w*_* (g mol^−1^) 5.6 × 10^−5^ T*_*m*_* (°C) 161 T*_*g*_* (°C) −3 T*_*c*_* (°C) 80	TS (MPa) 28.7 EB (%) 121.1 YM (MPa) 286	
P(3HB-*co*-7.5 mol% 3HV)		M*_*w*_* (g mol^−1^) 6.9 × 10^−5^ T*_*m*_* (°C) 174 T*_*g*_* (°C) −3 T*_*c*_* (°C) 70	TS (MPa) 19.0 EB (%) 13.6 YM (MPa) 272	
P(3HB-*co*-7.3 mol% 3HV)		M*_*w*_* (g mol^−1^) 7.1 × 10^−5^ T*_*m*_* (°C) 172 T*_*g*_* (°C) n.d. T*_*c*_* (°C) 60	n.d.	
P(3HB-*co*-5 mol% 3HV)	*A. hydrophila* 4AK4, grown on lauric acid and/or valerate	M*_*w*_* (g mol^−1^) n.d. T*_*m*_* (°C) 170 T_g_ (°C) 2.2	TS (MPa) 18.1 EB (%) 2.3 YM (MPa) 1465	Zhang et al., 2009
P(3HB-*co*-6 mol% 3HV)	*Cupriavidus necator*, odd-numbered carboxylic acid mix (FAM) as carbon source for large scale production of copolymers	M*_*w*_* (g mol^−1^) 4.4 × 10^5^ T*_*m*_* (°C) 159 T_g_ (°C) 2.2 T*_*c*_* (°C) 50.9	n.d.	Koller et al., 2014
P(3HB-*co*-9 mol% 3HV)		M*_*w*_* (g mol^−1^) 4.5 × 10^5^ T*_*m*_* (°C) 159 T*_*g*_* (°C) 3.3 T*_*c*_* (°C) 56	n.d	
P(3HB-*co*-12 mol% 3HV)	*C. necator*, grown on waste glycerol and rapeseed hydrolysates	T*_*m*_* (°C) 155 T*_*g*_* (°C) −1.9 T*_*c*_* (°C) n.d.	n.d.	García et al., 2013
P(3HB-*co*-13.5 mol% 3HV)	*A. oremlandii*, biodegradation of linseed oil-based elastomeric film	M*_*w*_* (g mol^−1^) 2.3 × 10^5^ T*_*m*_* (°C) 152 T*_*g*_* (°C) −3.8 T*_*c*_* (°C) n.d.	n.d.	Pramanik et al., 2014
P(3HB-*co*-26 mol% 3HV)	*C. necator* DSM 545, multiple-pulse feeding with valeric acid	M*_*w*_* (g mol^−1^) 1.1 × 10^5^ T*_*m*_*^1^ (°C) 142 T*_*m*_*^2^ (°C) 156	n.d.	Gahlawat and Soni, 2017
P(3HB-*co*-59 mol% 3HV)	Recombinant *Escherichia coli*, functionalization of copolymer PHBV with ascorbic acid	T*_*m*_* (°C) 135	n.d.	Bhatia et al., 2019a
P(3HB-*co*-2.35 mol% 3HV)	*H. bluephagenesis;* engineering of TCA cycle by CRISP/cas9 approach for copolymer production from glucose	T*_*m*_* (°C) 166 T*_*g*_* (°C) −0.4	n.d.	Chen et al., 2019
P(3HB-*co*-4.01 mol% 3HV)		T*_*m*_* (°C) 163 T*_*g*_* (°C) −0.5	n.d.	
P(3HB-*co*-8.6 mol% 3HV)		T*_*m*_* (°C) 158 T*_*g*_* (°C) 3.1	n.d.	
P(3HB-*co*-11.7 mol% 3HV)		T*_*m*_* (°C) 156 T_g_ (°C) 2.3	n.d.	
P(3HB-*co*-15.2 mol% 3HV)		T*_*m*_* (°C) 158 T*_*g*_* (°C) 2.8	n.d.	
P(3HB-*co*-19.6 mol% 3HV)		T*_*m*_* (°C) 137 T*_*g*_* (°C) 0.5	n.d.	
P(3HB-*co*-11 mol% 4HB)	Recombinant *E. coli*, unrelated carbon sources	T*_*m*_* (°C) 131 T*_*g*_* (°C) −4.4	TS (MPa) 20 EB (%) 698	Li et al., 2010
P(3HB-*co*-18 mol% 4HB)		T*_*m*_* (°C) 130 T*_*g*_* (°C) −9.2	TS(MPa) 9.9 EB (%) 729	
P(3HB-*co*-38 mol% 4HB)	*C. necator* strain A-04, different ratios of fructose and 1,4-butanediol	M*_*w*_* (g mol^−1^) 1.0 × 10^5^ T*_*m*_* (°C) 152 T*_*g*_* (°C) −10 T*_*c*_* (°C) 55	TS (MPa) 2.9 EB (%) 48 YM (MPa) 0.6 × 10^3^	Chanprateep et al., 2010
P(3HB-*co*-5 mol% 4HB)		M*_*w*_* (g mol^−1^) 1.4 × 10^5^ T*_*m*_* (°C) 161 T*_*g*_* (°C) −5 T*_*c*_* (°C) 83	TS (MPa) 0.9 EB (%) 22 YM (MPa) 0.8 × 10^3^	
P(3HB-*co*-24 mol% 4HB)		M*_*w*_* (g mol^−1^) 1.0 × 10^5^ T*_*m*_* (°C) 168 T*_*g*_* (°C) −2 T*_*c*_* (°C) 104	TS (MPa) 1.4 EB (%) 11 YM (MPa) 1.4 × 10^3^	
P(3HB-*co*-95 mol% 4HB)	*C. malaysiensis*, batch	M*_*w*_* (g mol^−1^) 4.4 × 10^5^	TS (MPa) 23 EB (%) 463 YM (MPa) 187	Norhafini et al., 2017
P(3HB-*co*-97 mol% 4HB)	*C. malaysiensis;* pulse feed of C and N sources	M*_*w*_* (g mol^−1^) 3.4 × 10^5^	TS (MPa) 31 EB (%) 473 YM (MPa) 272	
P(3HB-*co*-95 mol% 4HB)	*C. malaysiensis;* mixed feeding strategy	M*_*w*_* (g mol^−1^) 1.7 × 10^5^	TS (MPa) 24 EB (%) 471 YM (MPa) 214	
P(3HB-*co*-97 mol% 4HB)	*C. malaysiensis*; constant feeding strategy	M*_*w*_* (g mol^−1^) 3.2 × 10^5^	TS (MPa) 31 EB (%) 515 YM (MPa) 256	
P(3HB-*co*-99 mol% 4HB)	*C. malaysiensis;* twice pulse feed of C and N sources	M*_*w*_* (g mol^−1^) 3.3 × 10^5^	TS(MPa) 25 EB (%) 368 YM (MPa) 184	
P(3HB-*co*-20 mol% 4HB)	Recombinant, *Cupriavidus* sp. USMAA1020	M*_*w*_* (g mol^−1^) 391 × 10^3^ T*_*m*_* (°C) 129 T*_*g*_* (°C) −16	TS (MPa) 12 EB (%) 353 YM 72	Syafiq et al., 2017
P(3HB-*co*-85 mol% 4HB)		M*_*w*_* (g mol^−1^) 87 × 10^3^ T*_*m*_* (°C) 63 T*_*g*_* (°C) −41	TS (MPa) 11 EB (%) 380 YM(MPa) 103	
P(3HB-*co*-91 mol% 4HB)		M*_*w*_* (g mol^−1^) 60 × 10^3^ T*_*m*_* (°C) 53 T*_*g*_* (°C) −53	TS (MPa) 14 EB (%) 402 YM(MPa) 93	
P(3HB-*co*-10 mol% 4HB)	*C. necator* B10646, valeric acid, hexanoic acid, and γ-butyrolactone as precursor for copolymer production	M*_*w*_* (g mol^−1^) 5.7 × 10^5^ T*_*m*_* (°C) 150 T*_*g*_* (°C) 3.4 T*_*c*_* (°C) 66	TS (MPa) 14.8 EB (%) 5.7 YM (MPa) 957	Zhila and Shishatskaya, 2018
P(3HB-*co*-29 mol% 4HB)		M*_*w*_* (g mol^−1^) 8.3 × 10^5^ T*_*m*_* (°C) 162 T*_*g*_* (°C) n.d. T*_*c*_* (°C) 96	TS (MPa) 7.8 EB (%) 31 YM (MPa) 243	
P(3HB-*co*-75 mol% 4HB)		M*_*w*_* (g mol^−1^) 7.0 × 10^5^ T*_*m*_* (°C) 158 T*_*g*_* (°C) n.d T*_*c*_* (°C) 88	TS (MPa) 15.4 EB (%) 323 YM (MPa) 425	
P3HP	Recombinant *E. coli*, grown on mixtures of 1,3- propanediol (PDO) and 1,4-butanediol (BDO)	M*_*w*_* (g mol^−1^) 1.6 × 10^5^ T*_*m*_* (°C) 78 T*_*g*_* (°C) −18	TS (MPa) 22 EB (%) 498 YM (MPa) 2889	Meng et al., 2012
P(3HP-*co*-38 mol% 4HB)		M*_*w*_* (g mol^−1^) 2.8 × 10^5^ T*_*m*_* (°C) 63 T*_*g*_* (°C) −36	TS (MPa) 0.5 EB (%) 1611 YM (MPa) 4.4	
P(3HP-*co*-82 mol% 4HB)		M*_*w*_* (g mol^−1^) 3.0 × 10^5^ T*_*m*_* (°C) 36 T*_*g*_* (°C) −29	TS (MPa) 6.3 EB (%) 595 YM (MPa) 18.5	
P3HP	Recombinant *E. coli* for the synthesis of a block copolymer consisting of highly elastic P4HB portion with a P3HP block, PDO-BDO alternate feeding	M*_*w*_* (g mol^−1^) 1.6 × 10^5^ T*_*m*_* (°C) 78 T*_*g*_* (°C) −18	TS (MPa) 21 EB (%) 498 YM (MPa) 2889	Tripathi et al., 2013b
P4HB		M*_*w*_* (g mol^−1^) 3.9 × 10^5^ T*_*m*_* (°C) 61 T*_*g*_* (°C) −47	TS (MPa) 35 EB (%) 697 YM (MPa) 181	
P(3HP-*co*-25 mol% 4HB)		M*_*w*_* (g mol^−1^) 2.6 × 10^5^ T*_*m*_* (°C) 63 T*_*g*_* (°C) −31	TS (MPa) 6.4 EB (%) 963 YM (MPa) 15.5	
P(3HP-*co*-38 mol% 4HB)		M*_*w*_* (g mol^−1^) 2.8 × 10^5^ T*_*m*_* (°C) 63 T*_*g*_* (°C) −36	TS (MPa) 0.6 EB (%) 1611 YM (MPa) 4.4	
P3HP-b-29 mol% P4HB		M*_*w*_* (g mol^−1^) 5.5 × 10^5^ T*_*m*_* (°C) 55;68 T*_*g*_* (°C) −20; −46	TS (MPa) 45 EB (%) 877 YM (MPa) 177	
P3HP-b-37 mol% P4HB		M*_*w*_* (g mol^−1^) 5.5 × 10^5^ T*_*m*_* (°C) 53;67 T*_*g*_* (°C) −22; −45	TS (MPa) 25.3 EB (%) 1031 YM (MPa) 113	
P(3HB-*co*-8 mol% 3HV)	Wild-type *A. hydrophila*, using lauric acid as carbon source	T*_*m*_* (°C) 170 T*_*g*_* (°C) 2	n.d.	Noda et al., 2005
P(3HB-*co*-12 mol% 3HHx)		T*_*m*_* (°C) 110 T*_*g*_* (°C) −2.5	n.d.	
P(3HB-*co*-8 mol% 3HHx)		T*_*m*_* (°C) 140 T*_*g*_* (°C) 0	n.d.	
P(3HB-*co*-2.7 mol% 3HHx)	*C. necator*, regulation of 3HHx mol% by expression of R-specific enoyl-CoA hydratases	T*_*m*_* (°C) 151 T_g_ (°C) −2.3 T*_*c*_* (°C) 50	n.d.	Arikawa et al., 2016
P(3HB-*co*-5.9 mol% 3HHx)		T*_*m*_* (°C) 139 T_g_ (°C) −2.4 T*_*c*_* (°C) –	n.d.	
P(3HB-*co*-7.9 mol% 3HHx)		T*_*m*_* (°C) 131 T_g_ (°C) −2.2 T*_*c*_* (°C) –	n.d.	
P(3HB-*co*-10.8 mol% 3HHx)		T*_*m*_* (°C) 113 T_g_ (°C) −4.7 T*_*c*_* (°C) –	n.d.	
P(3HB-*co*-7 mol% 3HHx)	Crystallization study on commercial PHBPHHx (Kaneka corporation) containing different mol% HHx monomer	M*_*w*_* (g mol^−1^) 2.3 × 10^5^ T*_*m*_* (°C) 122; 141 T_g_ (°C) 2.3 T*_*cc*_* (°C) 62	n.d.	Cai and Qiu, 2009
P(3HB-*co*-10 mol% 3HHx)		M*_*w*_* (g mol^−1^) 4.5 × 10^5^ T*_*m*_* (°C) 121;139 T*_*g*_* (°C) 2.9 T*_*cc*_* (°C) 58	n.d.	
P(3HB-*co*-18 mol% 3HHx)		M*_*w*_* (g mol^−1^) 2.6 × 10^5^ T*_*m*_* (°C) n.d. T*_*g*_* (°C) 1.3 T*_*cc*_* (°C) n.d.	n.d.	
P(3HB-*co*-22 mol% 3HHx)	Recombinant *A. hydrophila*	M*_*w*_* (g mol^−1^) 2.9 × 10^5^	n.d.	Tian et al., 2005
P(3HB-*co*-3HO)	Enriched culture of *Pseudomonas* sp.; production of PHA from acidified oil mill wastewater	M*_*w*_* (g mol^−1^) 49 × 10^4^ T*_*m*_* (°C) 150; 163 T*_*g*_* (°C) 0.4	n.d.	Ntaikou et al., 2014
mcl copolymer from heptadecanoic acid	*P. aeruginosa* ATCC 27853, odd-chain fatty acids from C17 to C21 under nitrogen starvation	M*_*w*_* (g mol^−1^) 77 × 10^−3^ T*_*m*_* (°C) 52 T*_*g*_* (°C) −45	n.d.	Impallomeni et al., 2018
mcl copolymer from non-adecanoic acid		M*_*w*_* (g mol^−1^) 97 × 10^−3^ T*_*m*_* (°C) 48 T*_*g*_* (°C) −43	n.d.	
mcl copolymer from heneicosanoic acid		M*_*w*_* (g mol^−1^) 188 × 10^−3^ T*_*m*_* (°C) 49 T*_*g*_* (°C) −39	n.d.	
P(16 mol%3HD-*co*-3HDD)	*P. putida* mutant, grown on dodecanoate	M*_*w*_* (g mol^−1^) 15.5 × 10^4^ T*_*m*_* (°C) 78 T*_*g*_* (°C) −32	TS (MPa) 5.2 EB (%) 88 YM (MPa) 103	Liu et al., 2011b
100% 3HD		M*_*w*_* (g mol^−1^) 36.1 × 10^4^ T*_*m*_* (°C) 72 T*_*g*_* (°C) −37	TS (MPa) 12 EB (%) 313 YM (MPa) 20	
P(15 mol% 3HHx-*co*-40 mol% 3HO-*co*-30 mol% 3HD-*co*-15 mol% 3HDD)	*P. putida* mutant, sequential supplementation of hexanoate and dodecanoic acid	M*_*w*_* (g mol^−1^) 10 × 10^4^ T*_*m*_* (°C) 53 T*_*g*_* (°C) −44	TS (MPa) 8.7 EB (%) 189 YM (MPa) 3.6	Tripathi et al., 2013a
P(15.86 mol% 3HD-*co*-35.25 mol% 3HDD).		M*_*w*_* (g mol^−1^) 16 × 10^4^ T*_*m*_* (°C) 33;66 T*_*g*_* (°C) −43	TS (MPa) 16 EB (%) 369 YM (MPa) 38	
P(3HDD-*co*-10 mol%3H9D)	*P. entomophila* with deficient β-oxidation pathway	M*_*w*_* (g mol^−1^)9.5 × 10^4^ T*_*m*_* (°C) 69 T*_*g*_* (°C) −48	TS (MPa) 4.0 EB (%) 221 YM (MPa) 51	Li et al., 2014
P(3HDD-*co*-40 mol%3H9D)		M*_*w*_* (g mol^−1^) 8.8 × 10^4^ T*_*m*_* (°C) 52 T*_*g*_* (°C) −54	TS (MPa) 3.5 EB (%) 206 YM (MPa) 20	
P(3HDD-*co*-52 mol%3H9D)		M*_*w*_* (g mol^−1^) 1.0 × 10^5^ T*_*m*_* (°C) 45 T*_*g*_* (°C) −55	TS (MPa) 2.2 EB (%) 124 YM (MPa) 3	
P(3HDD-*co*-77 mol%3H9D)		M*_*w*_* (g mol^−1^) 9.9 × 10^4^ T*_*m*_* (°C) 43 T*_*g*_* (°C) −55	TS (MPa) 3.6 EB (%) 173 YM (MPa) 3	
P(3HDD-*co*-81 mol%3H9D)		M*_*w*_* (g mol^−1^) 9.4 × 10^4^ T*_*m*_* (°C) 43 T*_*g*_* (°C) −56	TS (MPa) 3.7 EB (%) 105 YM (MPa) 4.7	
P3HDD-b-70 mol%P3H9D		M*_*w*_* (g mol^−1^) 13 × 10^4^ T*_*m*_* (°C) 46 T*_*g*_* (°C) −55	TS (MPa) 3 EB (%) 138 YM (MPa) 8	
P(3HDD-*co*-29 mol% 3HD-*co*-12 mol% 3HTD-*co*-10 mol%3HO-*co*-6 mol%3HHx)	*Ps. chlororaphis subsp. aurantiaca*, using crude glycerol from biodiesel production as the sole carbon source	M*_*w*_* (g mol^−1^) 1.1 × 10^5^ T*_*m*_* (°C) 43 T*_*g*_* (°C) −47	TS (MPa) 3.9 EB (%) 273 YM (MPa) 8	Pereira et al., 2019
mcl-PHA (75 mol% 3HD)	Mixed culture of *Pseudomonas aeruginosa, Pseudomonas* sp., and *Ralstonia* sp.	T*_*m*_* (°C) 50; 82 T*_*g*_* (°C) −38 T*_*c*_* (°C) 20	n.d.	Sangkharak et al., 2020
mcl-PHA (75 mol% 3HD)/TGCN		T*_*m*_* (°C) 50; 82 T*_*g*_* (°C) −37 T*_*c*_* (°C) 12	n.d.	
P(3HDD-co-12 mol% 3HTD-co-10 mol% 3HO-co-6 mol% 3HHx)	mcl-PHA by *Pseudomonas mendocina* CH50 from waste oils; plasticising effect of the oligomeric mcl-PHA on P(3HB)	M*_*w*_* (g mol^−1^) 21 × 10^4^	n.d	Lukasiewicz et al., 2018
OligoHA: hydrolyzed P(3HHx-3HO-3HD-3HDD)		M*_*w*_* (g mol^−1^) 10 × 10^3^	n.d.	
P3HB-OligoHA blend (95/5)		T*_*m*_* (°C) 172	TS (MPa) 12.5 EB (%) 4 YM (MPa) 1000	
P3HB-OligoHA blend (90/10)		T*_*m*_* (°C) 175	TS (MPa) 15 EB (%) 6 YM (MPa) 1200	
P3HB-OligoHA blend (80/20)		T*_*m*_* (°C) 173	TS (MPa) 6 EB (%) 16 YM (MPa) 480	
P3HB		T*_*m*_* (°C) 177	TS (MPa) 20 EB (%) 5 YM (MPa) 1440	
P(3HDD*-co-*3HPhV)	*P. entomophila* grown on 5-phenylvaleric acid (PVA) and dodecanoic acid for synthesis of controllable composition of 3HDD and phenyl group on the side chain	M*_*w*_* (g mol^−1^) 10 × 10^4^ T*_*m*_* (°C) 82 T*_*g*_* (°C) −49	TS (MPa) 5.5 EB (%) 60 YM (MPa) 61	Shen et al., 2014
P(3HDD*-co-*2.9 mol% 3HPhV)		M*_*w*_* (g mol^−1^) 6.6 × 10^4^ T*_*m*_* (°C) 81 T*_*g*_* (°C) −33	TS (MPa) 2.0 EB (%) 37 YM (MPa) 94	
P(3HDD*-co-*18.7 mol% 3HPhV)		M*_*w*_* (g mol^−1^) 7.3 × 10^4^ T*_*m*_* (°C) 80 T*_*g*_* (°C) −36	TS (MPa) 4.4 EB (%) 86 YM (MPa) 95	
P(3HDD*-co-*32 mol% 3HPhV)		M*_*w*_* (g mol^−1^) 6.1 × 10^4^ T*_*m*_* (°C) 76 T*_*g*_* (°C) −35	TS (MPa) 3.1 EB (%) 32 YM (MPa) 49	
P(3HPhV)		M*_*w*_* (g mol^−1^) 4.41 × 10^4^ T*_*m*_* (°C) 50 T*_*g*_* (°C) 6	TS (MPa) n.d. EB (%) n.d. YM (MPa) n.d.	
P(3HB-*co*-11 mol% 3HV-*co*-10 mol% 3HHx)	Recombinant *A. hydrophila* 4AK4, grown on lauric acid, and sodium valerate	n.d.	TS (MPa) 8.4 EB (%) 341 YM (MPa) 235	Zhang et al., 2009
P(3HB-*co*-17 mol% 3HV-*co*-10 mol% 3HHx)		n.d.	TS (MPa) 14 EB (%) 740 YM (MPa) 97	
P(3HB-*co*-13 mol% 3HV-*co*-15 mol% 3HHx)		n.d.	TS (MPa) 13 EB (%) 833 YM (MPa) 66	
P(3HB-*co*-10 mol% 3HV-*co*-38 mol% 4HB)	*Cupriavidus eutrophus* B10646, valeric acid, hexanoic acid, and γ-butyrolactone as precursor for copolymer production	M*_*w*_* (g mol^−1^) 4.9 × 10^5^ T*_*m*_* (°C) 164 T*_*g*_* (°C) −5 T*_*c*_* (°C) 61	TS (MPa) 5.3 EB (%) 130 YM (MPa) 48	Zhila and Shishatskaya, 2018
P(3HB-*co*-17 mol% 3HV-*co*-55 mol% 4HB)		M*_*w*_* (g mol^−1^) 5.4 × 10^5^ T*_*m*_* (°C) 166 T*_*g*_* (°C) n.d. T*_*c*_* (°C) 25	TS (MPa) 8.8 EB (%) 365 YM (MPa) 34	
P(3HB-*co*-21 mol% 3HV-*co*-13 mol% 4HB-*co-*2 mol% 3HHx)		M*_*w*_* (g mol^−1^) 7.9 × 10^5^ T*_*m*_* (°C) 169 T*_*g*_* (°C) −0.7 T*_*c*_* (°C) 51	TS (MPa) 7.3 EB (%) 94 YM (MPa) 128	
P(3HB-*co*-6 mol% 3HV-*co*-9 mol% 4HB-*co-*1 mol% 3HHx)		M*_*w*_* (g mol^−1^) 7.6 × 10^5^ T*_*m*_* (°C) 161 T*_*g*_* (°C) −4.4 T*_*c*_* (°C) 63	TS (MPa) 12 EB (%) 49 YM (MPa) 419	

#### Poly (3-Hydroxybutyrate-co-Hydroxyvalerate)

Poly (3-hydroxybutyrate-*co*-hydroxyvalerate) is one of the most studied copolymers, has been developed on an industrial scale (Chang et al., 2014), and has recently attracted the attention of both industry and researchers as a promising material due to its biotechnological potentiality and its applicability in the medical, agricultural, and packaging fields (Rivera-Briso and Serrano-Aroca, 2018). PHBV has gained attention due to its better flexibility, strength, reduced chain packaging, and toughness compared to PHB (Tebaldi et al., 2019). The higher is the amount of the HV fraction, the lower is the melting point of the resulting PHBV copolymer, which broadens the processing window of the material (Ishida et al., 2005; Wang et al., 2013b). In addition, due to the longer side chain of HV, the segmental mobility in the amorphous phase of this copolymer increases, thus reducing the Tg (Ishida et al., 2005). Ultimately, PHBV copolymers show improvement in flexibility and ductility in comparison to PHB (Wang et al., 2013b).

PHBV thermomechanical properties vary widely depending on the mol% of 3-hydroxyvalerate (3HV). Although the incorporation of 3HV moieties in the PHB polymer is expected to reduce its crystallinity, this reduction is limited by the occurrence of the isodimorphism phenomenon, by which 3HB and 3HV are able to co-crystallize (Yeo et al., 2018). As a fact, PHBV still exhibits a high degree of crystallinity throughout a wide range of compositions from 0 to 95 mol% HV (Cai and Qiu, 2009).

Different cultivation strategies have been designed for the incorporation of 3HV monomers in the forming polymeric chain, using organic acid precursors, such as propionic and valeric acid (Zinn et al., 2003; Masood et al., 2012; Follonier et al., 2014; Hilliou et al., 2016; Martla et al., 2018).

In one of the first examples Madden et al. (1998) applied an alternate feeding strategy of glucose and propionic acid to *Ralstonia eutropha* cultures, to produce mixtures of PHB and smaller amount of 3HV-rich (7–18 mol% HV) random copolymer, PHBV. The process of chain termination resulted in the synthesis of polymer mixtures rather than block copolymers.

The demand for cost-competitive PHA production processes has translated into an increasing number of examples reporting the production of PHBV from waste materials and/or less costly, renewable precursors of HV moieties. Gahlawat and Soni (2017) tested the feeding with different acids to *Cupriavidus necator* cultures growing on waste glycerol from jatropha oil as the main carbon source. A multiple-pulse feeding strategy was applied to prevent growth inhibition caused by high acid concentration. In the best conditions, up to 25% HV content was achieved in the copolymer, reaching around 5 g/L polymer accumulation. The polymers produced in these conditions display a low molecular weight, consistently with the role of glycerol as chain termination agents. Similar production levels, although with a lower HV content (2.8–8%) were obtained by García et al. (2013) using all crude by-products (waste glycerol and rapeseed hydrolysates) as C-sources. A different approach to improve PHBV productivity as well as HV content (up to 36.7%) was applied by de Paula et al. (2017), through the selection of a *Pandoraea* sp. MA03 mutant strain with the highest performances on crude glycerol supplemented with propionic and valeric acids. In all the cases, the copolymers composition was found to be affected by the specific precursor feed rate, resulting into PHBV with different 3HV mol%. Saturated fatty acids from the animal processing industry were used as main C-source for the synthesis of PHBV copolymer from *C. necator* (Koller et al., 2014).

In view of process sustainability, the use of levulinic acid (LA) as 3HV precursor has been suggested by several authors. LA can be produced from a wide range of renewable biomaterials, such as cellulose-containing agricultural and forest wastes, in a cost-effective manner. Although *R. eutropha* could grow in the presence of LA as the sole carbon source, accumulation of PHBV occurred in significant amount only in the presence of glucose as cosubstrate and was strongly affected by the type of added nitrogen source (Wang et al., 2013b). More recently, the fine engineering of LA catabolic pathway in *Pseudomonas putida* EM42 allowed the production of various type of scl-PHA, containing 3HV and 4-hydroxyvalerate (4HV) with different monomer compositions and proportions, depending on the expression of different PHA reductases and synthases endowed with different substrate specificities (Cha et al., 2020). The incorporation of 4HV in place of 3HV into PHBHV has been reported to further decrease the melting point of the final copolymer (Schmack et al., 1998) and improve toughness without affecting the degradation temperature (Sheu et al., 2018).

Hydrolysates of residual spent coffee ground after oil extraction are also interesting source of LA, promoting the accumulation of PHBV copolymer (up to 11.6 mol% HV) by *Burkholderia cepacia* (Obruca et al., 2014). Another original example of waste valorization has been described by Pramanik et al. (2014), who reported the production of PHBV (13.8 mol% 3HV) from an alkaliphilic microbe, *Alkaliphilus oremlandii* OhILAs strain, through the biodegradation of linseed oil-based elastomeric film.

To overcome the need for acid cosubstrates, which cannot only inhibit cell growth but also increase production costs, PHBV copolymer synthesis has been also approached using glucose as C-source. To this aim, fine engineering strategies, focused on the pathways responsible for propionyl-CoA synthesis in *Haloferax mediterranei*, have been applied (Chen et al., 2011; Tan et al., 2014a; Yang et al., 2014). In one of the most recent examples, the CRISP/cas9 approach was used to simultaneously target different genes of the tricarboxylic acid cycle without compromising cell growth, resulting in up to 25 mol% 3HV in the synthesized polymer (Chen et al., 2019).

PHBV copolymers have also been produced in MMC-based processes, starting from a various range of complex organic substrates, such as food waste (Gouveia et al., 2017), municipal wastewater sludge (Wijeyekoon et al., 2018), the liquid fraction resulting from pyrolysis processes (Bio-oil) (Moita Fidalgo et al., 2014), and brewery wastewaters (Tamang et al., 2019). In an interesting example, the modulation of pH in the range of 4.5–7 during the acidogenic reactor operating conditions led to different monomer precursor profiles, which resulted in PHBHV copolymers with different compositions starting from cheese whey as model feedstock (Gouveia et al., 2017). By increasing the operating pH of the acidogenic reactor, up to 30 mol% HV could be achieved, while at low pH (<6), the HV content significantly reduced to 5 mol%. Similarly, Huang et al. investigated the effect of pH of β-cyclodextrin and glycerol on the profile of odd C-number VFA in the anaerobic digestion of waste-activated sludge. The glycerol amount was found to be the predominant factor in regulating the odd VFA proportion, being the latter positively correlated to the mol% HV of the synthesized copolymers (Huang et al., 2018).

In another recent report, a valerate-dominant sludge hydrolysate was fed to enrich a PHA culture under feast–famine conditions. The valerate uptake was shown to correlate with the production of 3HV and 3-hydroxy-2-methylvalerate (3H2MV) precursors, resulting in a P(3HB-co-23.7% 3HV-co-7.9% 3H2MV) mmol C % copolymer. Microbial analysis revealed that such valerate-rich feedstock caused *Delftia* to be the prevailing group over other PHA-producing bacteria (Hao et al., 2017).

Furthermore, the use of phototrophic mixed cultures (PMCs) for PHBHV production has also been proposed to decrease operational costs, as these microorganisms are able to draw energy from sunlight and not require oxygen to produce ATP. Fradinho et al. (2019) applied an anaerobic permanent feast strategy to select for a PMC consortium able to regulate internal reducing power via PHA production. When the selected PMC was applied to fermented cheese whey, a PHBHV copolymer with 12 mol% HV was obtained, using light intensities corresponding to those of direct sunlight illumination and without any aeration requirement.

The PHBV properties as a function of HV content (Table 2) indicate a gradual decrease in the Tm together with an improvement in EB, resulting in a range of polymers with enlarged processability, useful to make films and fibers with different elasticities (Anjum et al., 2016; Keskin et al., 2017). Biopol is the trade name of a PHBV copolymer, currently produced by Metabolix, having a range of uses such as packaging, disposable cutlery, razors, cups, shampoo bottles, as well as surgical stiches, pins, and medical patches (Anjum et al., 2016). Evidencing good gas barrier properties, this polymer can be a good candidate for the production of biodegradable food packaging material, able to both extend the shelf life of food products and delay the foods' spoilage. The physical–chemical and structural stability of PHBHV (3 mol% HV) films under food contact conditions was evaluated by Chea et al. (2016). It was concluded that mechanical properties and water vapor permeability of PHBHV films were preserved after contact at 40°C for 10 days with different food simulating liquids tested (water, acetic acid 3% w/v, ethanol 20% w/v, iso-octane) except with ethanol 95% (v/v).

Processing of PHBHV material via traditional melt spinning is hampered by the transition from viscoelasticity to brittleness that occurs with increase in storage time (Wang et al., 2016). The obtainment of PLA/PHBHV fibers via conventional melt-spinning and hot-drawing processes represented a solution to overcome this problem (Li et al., 2015a). Mechanical properties of PHBHV fibers were also improved through the application of drawing and heat settling processes after the formation of the fibers (Hufenus et al., 2015).

The use of PHBHV electrospun fibers to develop active multilayer materials for food packaging application was also investigated (Castro-Mayorga et al., 2017a; Figueroa-Lopez et al., 2020). An active PHBHV-based multilayer, loaded with silver nanoparticles, was found effective against *Salmonella enterica*. The mechanical performance of the PHBHV was not altered by the incorporation of the electrospun PHBHV coating, and the presence of AgNPs did not affect the properties of the coated system as well (Castro-Mayorga et al., 2017a). In a more recent example, a novel multilayer system based on PHBHV (2–3 mol% HV) with antimicrobial properties was developed. Electrospun mats of PHBHV fibers containing eugenol were used as an active interlayer between a food contact layer of PHBHV and a cast-extruded PHB sheet. After the annealing at mild temperature, the novel multilayer active packaging material exhibited, besides antimicrobial activity, high hydrophobicity, strong mechanical resistance, and improved barrier properties against water vapor and limonene vapors (Figueroa-Lopez et al., 2020).

Additional updated examples of PHBV bioactive nanocomposites will be detailed in section *PHB-Based Bionanocomposites*.

The uses of PHBHV copolymers have been also investigated in biomedical sectors, where they have been shown to provide a positive effect on cellular growth and adhesion (Keen et al., 2007; Ahmed et al., 2010). Furthermore, the more amorphous structure displayed by PHBV copolymer has favored its exploitation in drug delivery applications due to easy diffusion of active molecules. Despite the improved polymer properties, copolymers often exhibit slow degradability and resorbability due to their intrinsic hydrophobicity, which limits cell colonization. Bhatia et al. (2019b) addressed this issue through the functionalization of PHBV copolymer (59% HV) with ascorbic acid, mediated by *Candida antarctica* lipase B. The modified polymer displayed antioxidant activity as well as a 1.6-fold increase in biodegradability as compared to the neat copolymer. In addition, the functionalization also resulted into a lower degree of crystallinity (due to imperfection of crystals) and higher thermal degradation temperature and hydrophilicity degree.

#### Poly (3-Hydroxybutyrate-co-Hexanoate)

Poly (3-hydroxybutyrate-*co*-hexanoate) (PHBHHx) is a promising copolymer based on 3HB with minor contents of 3HHx comonomer. Differently from PHBV, the presence of short chain branches of three carbon atoms in PHBHHx has a marked effect on reducing the regularity of the polymer chain, thus lowering both crystallinity and Tm (Noda et al., 2010). Actually, although the crystallization mechanism and the crystal cell structure do not change, the overall isothermal crystallization rate of PHBHHx copolymers reduces with HHx content and occurs at lower crystallization temperature from the melt (Cai and Qiu, 2009). Indeed, due to their steric hindrance, the 3HHx does not co-crystallize with 3HB units; thus, PHBHHx displays slower crystallization rate than PHB homopolymers, which can be a challenge for its efficient processing (Vandewijngaarden et al., 2016). The fact is that due to reduced crystallinity, when PHBHHx copolymers with different mol% HHx are subjected to anaerobic biodegradation, the higher is the HHx, the faster is the rate of weight loss (Morse et al., 2011).

The synthesis of PHBHHx copolymer has been mainly obtained through strain engineering approaches, focusing on the specificity of the key PHA biosynthetic enzymes toward HHx precursors. The mol% HHx in PHBHHx copolymer was finely regulated (from 2.7 to 10.8%) by acting on the expression level of the *phaJ*s in a *C. necator* strain harboring the PHA synthase gene from *Aeromonas caviae*. The same gene target was also overexpressed in *R. eutropha* Re2133, together with a PhaC2 synthase from *Rhodococcus aetherivorans* endowed with a broad specificity for mcl-monomer, as well as with the deletion of *phaB* genes. This strategy was effective in promoting up to 22% HHx using coffee waste oil as the substrate (Bhatia et al., 2018).

*Escherichia coli* has been used as chassis for the production of PHBHHx by introducing synthase genes specific for mcl-precursors and/or acting on the pathways channeling specific precursors (Taguchi et al., 1999; Lu et al., 2003). A high mol% HHx (50 mol%) has been achieved in *E. coli* strain engineered with the PHA biosynthetic operon from *Bacillus cereus*, using fatty acids as well as complex C-sources such as waste frying oils as substrates (Vastano et al., 2017, 2019).

Most of the engineering approaches have been focused on *Aeromonas hydrohila*, this strain being naturally able to produce PHBHHx from dodecanoate. First attempts of strain engineering reveal the synergic effect of the overexpression of phasins—proteins associated to PHA granules in the cells—and PhaJ coding genes in increasing 3HHx fraction (Han et al., 2004). An increase in 3HHx content is also obtained by deleting acetic acid pathway-related genes (Liu et al., 2011a). Interestingly, the overexpression of phasin coding genes in the engineered *A. hydrophila* strain not only determines an increase in 3HHx content but also causes a reduction in polymer molecular weight due to the formation of more PHA granules with reduced size (Tian et al., 2005).

To address PHBHHx production from glucose or gluconate instead of fatty acids*, A. hydrophila* and *P. putida* have been engineered with different combinations of target genes, with tesA thioesterase and phaG being the key targets in the two strains, respectively (Qiu et al., 2005).

In terms of thermomechanical properties, PHBHHx combines those of polyethylene (PE) (i.e., strength, flexibility, toughness, and elasticity), with printability and dyeability features (Anjum et al., 2016). Compared to conventional polymers used in packaging, PHBHHx films obtained by compression molding display relatively low oxygen permeability and a water vapor permeability slightly higher than those of PE, PP, and PS. CO_2_ permeability is rather high if compared to known barrier materials (PET, PA, and EVOH) but also lower than those for packaging materials such as PP and PE (Vandewijngaarden et al., 2014). Despite its potential in food packaging application, the slow crystallization that characterizes this copolymer has represented an obstacle for its industrial processing. In fact, the addition of different additives acting as nucleating agents has been proposed. Ultrafine talc was reported to drastically improve PHBHHx crystallization, causing also an increase in the YM (Vandewijngaarden et al., 2016) without modification of the material barrier properties, thus opening the way to its use as a protection layer for moisture-sensitive O_2_ barrier layers. The use of zinc oxide as filler, on the other hand, also improved PHBHHx crystallization but strongly affected its opacity, although resulting in a successful UV-blocking property.

The exploitation of PHBHHx copolymers in combination with reinforcing materials (Dehouche et al., 2020) as well as in blending with other polyesters, mainly polylactic acid (PLA), can provide final materials with improved properties. Bio-composites based on PHBHHx and aloe vera fibers (AVFs) have been prepared by testing different surface treatment methods of AVF in order to improve the interfacial adhesion between the fiber and the polymer matrix (Dehouche et al., 2020). Blending of PHBHHx (11% HHx) with poly(DL-lactide) (PDLLA) from solvent casting, at ratios of 2:1 and 1:2, exhibit a lower YM and a higher EB compared to unblended PDLLA, whereas melt compounding of PLA and PHBHHx in different ratios inhibits PLA crystallization, resulting in enhanced elongation and toughness with respect to neat PLA (Lim et al., 2013).

Due to its biocompatibility and higher elasticity compared with PHB and PHBV, PHBHHX has been widely applied as scaffold matrix in tissue engineering and cell transplantation (Gao et al., 2006).

3HHx content affects *in vitro* growth and differentiation of smooth muscle cells (Qu et al., 2006a). When compared to PHB, PHBHHx reveals a higher degradation rate in subcutaneous implants in rabbits, and, most importantly, the copolymer elicits a very mild tissue response during implantation (Qu et al., 2006b). In one of the most recent examples, PHBHHx supports the residence, survival, and stemness of the transplanted neural stem cells (NSCs) cells in rat brain (Wang et al., 2019).

Surface modification of PHBHHx films under alkaline conditions has been found effective in promoting osteoblast cell response for application in bone-tissue engineering (Li et al., 2005). In a pioneering approach, Li et al. (2015b) have coated PHBHHx scaffolds with PHA binding protein (PhaP) fused with the arginyl-glycyl-aspartic acid peptide (PhaP-RGD) to promote proliferation and differentiation of mesenchymal stem cells seeded on them. Due to their reliable safety profile and strong hydrophobicity, PHBHHx is suitable for prolonged release delivery systems. Implantable sandwich PHBHHx films have been designed to address long time release of drugs, reducing the burst release effect (Peng et al., 2018). In addition, PHBHHx nanoparticles have been applied to this aim (Peng et al., 2013; Heathman et al., 2014). Interestingly, a hybrid copolymer, such as PEG200-end capped PHBHHx, has been synthesized by *A. hydrophila* in microbial fermentation and the derived nanoparticle tested as intracellular delivery nanocarriers for sustained drug release (Lu et al., 2004). Noteworthy, besides drug delivery, incorporation of PHBHHx nanoparticle in whey protein-based films, improves the mechanical properties of the derived bio-plastics producing more extensible materials preserving their mechanical resistance (Corrado et al., 2021).

#### Poly (3-Hydroxybutyrate-co-4-Hydroxybutyrate)

Several studies have been focused on the synthesis of poly (3-hydroxybutyrate-*co*-4-hydroxybutyrate) (P3HB4HB) copolymers, using wild-type strains under feeding of 4HB precursors such as 4-hydroxybutyric acid, γ-butyrolactone, 1,4-butanediol, etc. (Choi et al., 1999; Lee et al., 2004).

Copolymers with different mol% 4HB (from 5 to 64%) have been produced in *C. necator* strain A-04 by tuning the ratios of fructose to 1,4-butanediol. The characterization of the copolymers reveals an increase in both elongation at break and tensile strength, as well as a reduction in polymer toughness, with increasing 4HB content (Chanprateep et al., 2010).

Co-feeding of soybean oil and γ-butyrolactone produces high yield of copolymer accumulation, although higher supplementation of the precursor has been shown to increase the 4HB fraction while inhibiting cell growth (Park and Kim, 2011). High PHA density fed-batch cultivation strategies employing mixed precursors (5:1 ratios 1,4-butanediol and 1,6-hexanediol) have been developed to boost the 4HB content up to 99 mol% in *Cupriavidus malaysiensis* transformed with additional copies of PHA synthase gene (PhaC) (Norhafini et al., 2017, 2019; Syafiq et al., 2017).

Waste materials such as saccharose from sugarcane industry (de Sousa Dias et al., 2017) and waste frying oils (Rao et al., 2010) have been also used in combination with 4HB precursors in processes employing *Burkholderia sacchari* and *C. necator*, respectively. Accumulation of P3HB4HB from slaughterhouse residues was achieved by using *Delftia acidovorans* DSM39 expressing and heterologous lipase (Romanelli et al., 2014).

The high cost of the 4HB precursors supplied to the growth media has encouraged the construction of engineered strains able to produce P3HB4HB copolymer from unrelated C-sources. An engineered *E. coli* strain has been designed to produce up to 65.5% P3HB4HB (11.1 mol% 4HB) accumulation using glucose as C-source. To enhance the carbon flux to 4HB biosynthesis, genes involved in succinate formation from succinate semi-aldehyde (succinate semi-aldehyde dehydrogenase) have been deleted (Li et al., 2010). A significant increase in copolymer accumulation has been achieved by the “enlarged cell strategy,” based on the alteration of the genes involved in cell division, in order to get filamentary *E. coli* with larger internal space. As an additional advantage, this filamentary recombinant *E. coli* also allows easier downstream separation from the fermentation broth (Wang et al., 2014). A P4HB homopolymer (PHA4400) is currently commercialized by Tepha Inc. (Cambridge, MA), which uses a proprietary transgenic fermentation process based on *E. coli*. The polymer is characterized by remarkable flexibility and good absorbability behavior *in vivo*, being well-suited for implantable medical applications. Noteworthy, the first products approved by the Food and Drug Administration (FDA) for clinical uses were P3HB4HB-based devised produced by Tepha (US) (Zhila and Shishatskaya, 2018).

A low-cost platform for non-sterile and continuous production of P3HB4HB from glucose has been designed using the halophilic *Halomonas bluephagenesis* as chassis. In this example, combinatorial deletions of multiple orthologs of succinate semi-aldehyde dehydrogenases has allowed to increase the 4HB molar fraction up to 24-fold (Ye et al., 2018).

Finally, a complex engineering strategy has been applied to *E. coli* to get co-polyesters of 3-hydroxypropionate (3HP) and 4HB by redesigning the pathways for the synthesis of the corresponding monomers from 1,3-propanediol (PDO) and 1,4 butanediol (BDO). P(3HP-co-4HB) with adjustable monomer ratios have been produced and characterized (Meng et al., 2012). Interestingly, the same recombinant *E. coli* strain has been used for the synthesis of a block copolymer consisting of highly elastic P4HB portion together with a P3HP block endowed with enormous tensile strength, by applying an alternate PDO-BDO feeding. In comparison to the homopolymers P3HP and P4HB, the block microstructure displays reduced Tm as well as increased YM and TS (Tripathi et al., 2013b).

#### Medium Chain Length PHA Copolymers

The incorporation of longer monomers (C ≥6) within the PHA polymeric chain determines the shift toward a more elastomeric behavior of the resulting polymer. These properties suit well with applications in tissue engineering and drug delivery, justifying the great interest in this research area.

Most of mcl-PHA are produced from *Pseudomonas* sp., which possess the specific metabolic pathways necessary for the synthesis of mcl-precursors from fatty acids. Mcl-copolymers with monomer composition ranging from C5 to C17 are synthesized by *P. aeruginosa* ATCC 27853 grown on odd-chain fatty acids from C17 to C21 under nitrogen starvation. The highest yield is obtained for heptadecanoic acid, probably because of limitation in the uptake of longer fatty acids. The characterized polymers are soft, sticky, rubber-like materials (Impallomeni et al., 2018). Previously, mcl-PHA production has been achieved by supplying even-chain n-alkanoic acids from C8 to C22 (Ballistreri et al., 2001). The use of complex oily sources, including waste frying oils, has been also explored for the synthesis of mcl-PHA from different *Pseudomonas* strains (Haba et al., 2007; Gamal et al., 2013; Follonier et al., 2014; Vastano et al., 2019).

The comonomer composition can be finely regulated by using β-oxidation weakened mutants of *P. putida* (Wang et al., 2011), so that the monomer composition is strictly related to the nature of the supplied fatty acid (Liu et al., 2011b). In an interesting example, a β-oxidation *P. putida* mutant has been exploited for the production of a novel diblock copolymer P3HHx-b-P(3HD-co-3HDD) by sequential supplementation of hexanoate and dodecanoic acid (Tripathi et al., 2013a). The incorporation of block microstructure determines an increase in TS, YM, and EB if compared to corresponding random copolymers. Thus, the blocky feature helps to capture the amorphous nature of the mcl-polymers, as well as to gain crystallinity, improving the overall polymer properties (Tripathi et al., 2013a). The incorporation of unsaturated PHA site chains represents a useful strategy to obtain functional and easily modifiable PHAs. By using a *Pseudomonas entomophila* strain with deficient β-oxidation pathway, the incorporation of 3-hydroxydodecanoate (3HDD) and 3-hydroxy-9-decenoate (3H9D) moieties in different ratios has been obtained from feeding with mixture of dodecanoic acid (DDA) and 9-decenol (9DEO). Due to the presence of unsaturated bonds, the copolymers can be cross-linked under UV radiation. Moreover, a diblock polymer P3HDD-b-P3H9D has been synthesized under specific feeding strategies. This polymer displays a 2-fold increase in YM compared with the random copolymer with similar 3HDD/3H9D ratios (Li et al., 2014).

A rather unique copolymer composed of 3HB and 3HO units was obtained using an enriched culture of *Pseudomonas* sp. as initial inoculum for the production of PHA from acidified oil mill wastewater. The weight average molecular mass of polymer was characterized by a wide polydispersion index, as a consequence of the high complexity of the final microbial consortium (Ntaikou et al., 2014).

When the so-called glycogen-accumulating organisms (GAOs) from mixed culture anaerobic–aerobic wastewater treatment processes were used to produce PHAs from acetate, the applied operational conditions were found to affect polymer microstructure. As a fact, a mixture of true copolymers consisting of monomers HB, HV, 3H2MV, and 3-hydroxy-2-methylbutyrate (HMB) was produced under anaerobic conditions, while the aerobic process primarily produced the HB monomer, most likely forming separate homopolymer blocks (Dai et al., 2008).

Despite their potential, the widespread use of mcl-PHA is limited by the difficulty in their processing, which is due to low viscosity, poor melt strength, adhesive-sticky nature, and very slow crystallization. Gopi et al. (2018) have proposed a chemical modification strategy involving the reaction with dicumylperoxide and triallylotrimesate co-agent to introduce branching and thus enhancing the crystallization kinetics of poly(3-hydroxydecanoate) (P3HD). The authors pointed out a possible application of these improved polymers in 3D printing of biomedical products.

The adhesive properties of an mcl-PHA (43 mol% HDD, 12 mol% HTD, 10 mol% HO, 6 mol% HHx) produced by *Pseudomonas chlororaphis* subsp. *aurantiaca* when grown on crude glycerol were investigated by Pereira et al. (2019). The films displayed good adhesion properties toward porcine and human skins, together with high tension and shear bond strength, suggesting the potential use of this material as novel natural adhesive for wound dressing. A different applicative scenario for this class of polymers has been recently reported (Sangkharak et al., 2020). The authors designed a novel bio-composite of an mcl-PHA (75 mol% HD) incorporating torch ginger cellulose nanowhiskers (TGNCs) as a filtering material for wastewater treatment.

Finally, an mcl-PHA (7.6 mol% HHx, 45.4 mol% HO, 41.8 mol% HD, 5.2 mol% HDD) produced by *Pseudomonas mendocina* CH50 from waste oils was transformed into an oligomeric derivative by acid hydrolysis and efficiently used as a plasticizer for PHB, resulting in softer and more flexible materials. The obtained materials, entirely based on PHA, were found applicable in soft tissue engineering, thanks to their improved properties as well as the demonstrated *in vitro* biocompatibility (Lukasiewicz et al., 2018).

#### Other Copolymers

Manipulation of synthetic pathways for PHA production has allowed to design new engineered strains able to incorporate unnatural monomers within the polymer backbone (Choi et al., 2020). Whatever is the unnatural monomer to be introduced in the bio-polyester, the designing of new evolved PHA synthases endowed with proper specificity in recognizing uncommon substrates is *a sine qua non*-condition for effective *in vivo* synthesis (Park et al., 2012). The introduction of a phenyl group on the side chain has been obtained in *P. entomophila* β-oxidation weaken mutant, in the presence of 5-phenylvaleric acid (PVA) as precursor (Shen et al., 2014). A microbial cell factory for the production of P(LA-co-3HB) copolymer has been designed through fine *E. coli* engineering (Taguchi et al., 2008). Besides the PhaA and PhaB from *R. eutropha*, the recombinant strain expresses a propionyl-CoA transferase (PCT) from *Megasphaera elsdenii*, able to catalyze coenzyme A addition to lactic acid, and a mutated PHA synthase from *Pseudomonas* sp. 61-3, endowed with the ability to polymerize lactoyl-CoA precursor. Interestingly, the same strain was exploited for the production of P(LA-co-3HB) from on a lignocellulosic feedstock, taking advantage of the *E. coli* abilities to metabolize both xylose and galactose derived from woody-extract hemicellulosic hydrolysate (Takisawa et al., 2017).

A similar approach allows to incorporate 2,3-dyhydroxybutyrate (DHBA) in engineered *E. coli* using glycolate as the sole C-source (Insomphun et al., 2016).

Properly designed feeding strategies, combined with a set of PHA synthetic genes with suitable specificities, have been developed to obtain complex ter- or quarter-polymers. Terpolyesters composed of HB, HV, and HHx monomers have been produced by providing *Aeromonas hydrophila 4AK4* strain with a set of PHA synthesis genes, allowing to supply the corresponding monomer precursors from lauric acid and/or valerate. Among the different synthesized polyesters, P(3HB-*co*-11% 3HV-*co*-10% 3HHx; PHBV11HHx10), and P(3HB-*co*-17% 3HV-co-10% 3HHx; PHBV17HHx10) have the suitable combination of YM, EB, and TS ranging from 97 to 235 MPa, 341 to 740%, and 8.4 to 14.3 MPa, respectively (Zhang et al., 2009).

In a similar approach, *R. eutropha* has been engineered with *A. hydrophila* PHA biosynthetic operon to produce PHBV7HHx11 and PHBV18HHx11 when fed with increasing amount of propionic acid. Compared with PHBHHx with 12 mol% HHx (PHBHHx12), the terpolymer has higher crystallization rate and degree of crystallinity. Crystallization studies have revealed that the simultaneous introduction of 3HHx and 3HV monomers in PHB improves the mobility of chain stems along the chain direction, leading to easier intralamellar slip during heating or drawing, finally resulting in improvement of mechanical properties (Ye et al., 2010). Furthermore, different P3HB4HB biopolymers, P3HBHV4HB terpolymers, and P3HBHV4HBHHx quarter-polymers with varying 4HB amount have been synthesized by *C. necator* strains under specific feeding strategies. The effect of 4H monomer unit into all the synthesized copolymers is in lowering the melting and the crystallization temperatures, thus improving processing-related properties of these materials. All the copolymers also display enhanced EB compared to PHB (Zhila and Shishatskaya, 2018).

### Tuning Polymer Molecular Weight

The average molecular weight of PHB synthesized by bacteria is usually in the range of 0.1–2.0 × 10^6^ g mol^−1^ in *R. eutropha* (Tsuge, 2016). However, ultrahigh molecular weight PHB (UHMW-PHB) with defined Mw 43.0 × 10^6^ g mol^−1^ display high mechanical strength (TS, 1,320 MPa; EB, 35%; YM, 18.1 GPa) and are preferred in applications requiring high mechanical strength, such as in developing high-strength fibers and films (Iwata, 2005).

Several factors have been shown to increase molecular weight during *in vivo* synthesis of PHA polymers: the concentration and the activity of the PHA synthase, the occurrence of chain transfer (CT) reactions, and the simultaneous degradation of PHA during biosynthesis (Tsuge, 2016). The molecular weight has been shown to decrease with increasing PHA synthase concentration and to increase proportionally with its catalytic activity. A CRISPRi-based approach to modulate PhaC expression level in *E. coli* has come to the same correlation: the higher the PhaC activity, the more the PHA accumulation, yet the less the molecular weight and the wider the polydispersity (Li et al., 2017).

The CT reaction is caused by the presence of molecules (CT agent) that promote the transesterification reaction between their hydroxy group and the carboxy group of the growing polymer chain-PHA synthase complex. Commonly occurring CT agents include water, 3HB, and ethanol. Thus, high-molecular-weight PHA can be synthesized, for example, by ensuring sufficient culture aeration to facilitate cell growth while preventing ethanol production. Furthermore, deletion of PHA depolymerase genes promotes the production of high-molecular weight PHA by turning off the polymer degradation within the cell (Tsuge, 2016).

## Chemical and Physical Strategies to Improve *Post-Synthesis* PHB-Based Materials

### Use of Plasticizers

In general, in the field of polymers, it is possible to identify two types of plasticizers: internal and external (Vidéki et al., 2007). The internal plasticizers are part of the polymer molecules, as they are chemically bound to the polymer chains, grafted or reacted with the original polymer, thus making the polymer chains more difficult to adapt and compact together closely. Their main role is to soften the polymers by lowering their glass transition temperature (Tg) and reducing their elastic modulus (Vieira et al., 2011). External plasticizers, instead, are low volatility molecules added to interact with polymers, widening the polymer chains without chemical reaction. In this case, the internal molecular forces between plasticizer molecules and between a plasticizer and a polymer play a fundamental role, such as dispersion forces, induction forces, dipole–dipole interaction, and hydrogen bonds (Mekonnen et al., 2013). Several theories have been proposed to explain the mechanism and role of plasticizers in polymers. The most important are (a) the lubricity theory, (b) the gel theory, and (c) the free volume theory (Vieira et al., 2014).

Although these theories are widely accepted and used in plasticizer selection for polymers, Shtarkman and Razinskaya (1983) have emphasized the limitation of current theories on the mechanism of plasticization. Indeed, according to these authors, it is not possible to establish a plasticization mechanism because of the huge versatility of the investigated polymer and plasticizer systems; hence, instead of trusting on the above theories, it would be much more useful to consider the direct correlation between compatibility–efficiency–property of each investigated polymer–plasticizer blend. As an example, since the behavior of a single polymer highly depends on its thermal history, different film processing can provide substantial diverse crystalline pattern, and the effect of the same plasticizer on these polymer substrate will, therefore, be quite different.

Therefore, the choice of a plasticizer involves several important criteria, including a high degree of compatibility with the polymer matrix, responsible of its easy solubility and inclusion in both crystalline (toughening) and amorphous (softening) regions. The compatibility should be consistent with the whole temperature range of applications; hence, plasticizer molecular mass and chemical structure, including polarity, shape, and size, must be considered to assure a suitable plasticizing effect. Generally, plasticizers are responsible for Tg lowering and elastic modulus decreasing, as reported above. Hence, the efficiency may be expressed in terms of Tg depression and stiffness reduction. With the lowering of Tg, elongation at break and flexibility of the polymer increases. In fact, elongation and impact resistance strongly depend on polymer Tg and molecular structural organization. In addition, the plasticizers should remain inside the polymer matrix. The larger the plasticizer, the lower its vapor pressure, and thus, the greater its permanence inside the polymer; on the other hand, bigger molecules are slowly diffused inside the macromolecular chains, since the highest diffusion rate is associated to small molecules that, on contrary, show higher vapor pressures (Vieira et al., 2011).

As noted above, the blending with the plasticizers is considered to be one of the simplest route to overcome the limitations of PHB, allowing for a broader application window. Generally, the use of natural or non-natural plasticizers would allow to lower the glass transition and melting temperature, through the enhanced macromolecular movement. In this way, it would be possible to process the polymer at lower temperatures without inducing its thermal degradation (Baltieri et al., 2003; Erceg et al., 2005). Furthermore, plasticizers could improve both the toughness and softness of the polymer by decreasing its crystallinity, weakening the intramacromolecular bond, and facilitating conformational changes.

For these reasons, many efforts have focused on the industrial formulation of PHB with external plasticizers able to improve their thermal and mechanical properties (Mangeon et al., 2018). The relevant literature is rich in number and type of plasticizers for PHB. They vary from those of natural origin to synthetic one from those with low to high molecular weight, from linear to branched structures. Table 3 summarizes some of the best known plasticizers in the literature, together with the main properties investigated, when used in mixing with PHB with a molecular weight in the range of 100,000–300,000 g mol^−1^.

**Table 3:** Main plasticizers for PHB.

**Plasticizers**	**Acronyms**	**M_*w*_ (g mol^−1^)**	**% w/w**	**T_*g*_ (°C)**	**T_*m*_ (°C)**	**EB (%)**	**TS (MPa)**	**YM (MPa)**	**References**
Glycerol	G	92	13		170	0.9	24.8	2835	Jost and Langowski, 2015
Triacetyl glycerol	TAG	218	20	−16.7	161	10.3	14.1	238.4	Baltieri et al., 2003
Dioctyl phthalate	DOP		20	−9.4	164.5	10.5	18.0	448	Baltieri et al., 2003; Wang et al., 2008
Dioctyl adipate	DOA		30	−6.9	165.3	6.2	16.1	576	
Dioctyl sebacate	DOS		30	−3.6	163.5	4.3	6.0	374	
Propylene glycol	PG	76	17		171	0.9	24.9	3,360	Râpe et al., 2015
Triethyl acetate	TEC	276	20	−30	164	2.3	15.8	1,135	Choi and Park, 2004; Râpe et al., 2015
Soybean oil	SO	920	20	−3.4	161		15		Choi and Park, 2004
Epoxidized soybean oil	ESO	975	13–20	−19	160	1.0	30	2,729	Choi and Park, 2004; Jost and Langowski, 2015; Panaitescu et al., 2017
Castor oil	CO	933	13		171	0.9	27.1	3,089	Jost and Langowski, 2015
Triethyl citrate	TC	276	30	−24	–	21	18.6	960	Râpe et al., 2015
Tributyl citrate	BTC	515	30	−23	–	32	9.4	800	Râpe et al., 2015
Acetylbutyryltrihexyl citrate	ABTC	558	30	−30.7	156.8	9.7	6.1	192.7	Erceg et al., 2005; Râpe et al., 2015; Chaos et al., 2019
Salicylic acid decylester	–	–	30	−28	–	104	11.8	850	Kunze et al., 2002
Salicylic acid 2-butyloctyl ester	–	–	30	−28	–	22	13.5	810	Kunze et al., 2002
Acetylsalicylic acid hexylester	–	–	30	−14	–	126	11.8	680	Kunze et al., 2002
Ketoprofenethylester	–	–	30	−14	–	58	11.8	600	Kunze et al., 2002
polyhydroxy-butyrate-hexanoate	PHHBX	1760	PHB−1/6	−10	102	9.8	25		Wang et al., 2008
Polyethylene glycol	PEG 1000	950–1050	50	–	170	1.0	23	2,700	Bibe et al., 1999
Linalool	L	154	20	−11.0	174	5.2	20	560	Mangeon et al., 2018
Geraniol	G	154	20	−9.0	179	5.9	21	760	Mangeon et al., 2018
Geranyl acetate	GA	196	20	−13.0	170	13.8	15	395	Mangeon et al., 2018
Dodecanol		186	10	7	155				Yoshie et al., 2000
Lauric acid	–	200	10	4	171				Yoshie et al., 2000
Tributyrin	–	302	10	1	172				Yoshie et al., 2000
Trilaurin	–	639	10	−4	173				Yoshie et al., 2000

From Table 3, it emerges that blending with plasticizers induce a general lowering of the glass transition temperature and an improvement in elongation at break for almost all the additives used, with few exceptions. The difference can be ascribed to the variation in combining the chemical structure of the plasticizer, the molecular weight, the solubility, and compatibility with the polymer, as above discussed. Therefore, the summarized plasticizers can be discussed after their subdivision based on building block molecule: (i) glycerol, (ii) oil, (iii) citric acid, and (iv) salicylic acid. Besides these, we must also add the category of plasticizers that have structures similar to some phthalates, common plasticizers of PVC, and those of smaller natural molecules such as those of terpenes.

Yoshie et al. (2000) compared the effect of tributyrin, trilaurine, lauric acid, and dodecanol on the physical properties of PHB. All the molecules used act to reduce the Tg and the cold crystallization temperature, Tcc. In this trend, the best seems to be tributyrin, given its good miscibility with the polymer chains. Although there is this advantage, all additives, even in small amounts (1% by weight), promote the enzymatic degradation of the polymer.

Recently, bio-based compounds such as the terpenes have also been studied as additives for PHB, thanks to their additional beneficial properties such as antioxidant and antibacterial activities (Persico et al., 2012). Terpenes are interesting components of essential oils extracted from plants, with a chemical structure of repeating units of isoprene (C_5_H_8_). Among these, the oxygenated monoterpenes that contain alcohol have been described as having greater biological activity. Mangeon et al. (2018) studied linalool (L), geraniol (G), and geraniol acetate (GA) as PHB plasticizers. In this study, the use of terpenes led to a decrease in Tg and an increase in elongation at break over 650% combined with a decrease in Young's modulus compared to pure PHB. The effect is more pronounced with GA due to the presence of the segment bearing an ester group, which increases free volume and molecular mobility. The fact that terpenes are already widely used in the chemical industry gives them real potential as a PHB plasticizer with antibacterial properties.

Salicylic acid esters and ibuprofen ketones have also been reported as suitable plasticizers of PHB. Their use, given their properties, is recommended for medical packaging (Kunze et al., 2002).

Dibutyl sebacate (DBS), dioctyl sebacate (DOS), polyethylene glycol (PEG), and Lapro1503 (L503), have also been reported in the literature as biodegradable lower molecular weight plasticizers to improve the properties of PHB. All the additives proved to be compatible with the polymer, forming monophasic mixtures up to a concentration of 15–20% by weight. For all, a decrease in Tg and an improvement and a decrease in the crystallization temperature were found (Baltieri et al., 2003; Wang et al., 2008).

Citric acid ester plasticizers are among the most important plasticizers and environment friendly because of their safety and non-toxicity. They have been approved in the United States, the European Union, and other developed countries for use in plastic products in close contact with the human body and meet high hygiene requirements (Chabrat et al., 2012). Acetyl tributyl citrate (ATBC) and tributyl citrate (TBC) are among the most studied in detail, thanks to their excellent performance. In addition, TBC has antibacterial and flame-retardant properties, which further expand its applicability (Chaos et al., 2019).

Many of these have been described as good plasticizers for PHB, improving its processability, in particular ATBC, TBC, and triethyl citrate (TC). The incorporation of plasticizers into PHB decreased TS and YM but increased the elongation at break. TBC and ATBC were the most compatible and efficient plasticizers on improving the thermal, mechanical, and barrier properties of PHB. The optimum concentration could be up to 20% depending on the desired properties of the final products (Erceg et al., 2005; Râpe et al., 2015; Chaos et al., 2019). From the study of Choi and Park (2004), triethyl citrate was the most effective plasticizer in terms of reduction in the glass transition temperature as well as in terms of improvement in the impact strength and elongation.

This result can be explained by the decrease in crystallinity and the crystalline size and in the formation of small spherulites, due to the easier penetration of the additive molecules between the polymer chains, which reduce the hydrogen bond. These data were correlated with the increase in elongation at break of the initial degradation temperature of the PHB.

Suárez Palacios et al. (2014) postponed the use of glycerol-based plasticizers and as an alternative to phthalates in the medical field. When mixed with PHB, glycerol and its derivatives showed good results due to their polarity (Jost and Langowski, 2015).

Many works in the literature point out that the use of high molecular weight plasticizers has a more pronounced effect in improving the mechanical properties. This is the case with PEG and epoxidized oils (Bibe et al., 1999). Epoxidized oils are among the most studied plasticizers of PVC, obtained by epoxidation with peracids with various oils (Turco et al., 2019b). The behavior in mixture with PHB can be explained by the higher reactivity of the epoxide group and the possibility of hydrogen bond formation (Choi and Park, 2004; Jost and Langowski, 2015; Panaitescu et al., 2017).

The main drawback in the use of plasticizers mainly concerns the external plasticization with the migration of the plasticizer from the plasticized material. This process can occur by diffusion of the plasticizer from the bulk material to the surface (exudation) or by interface phenomena and absorption or by evaporation in the surrounding medium. This phenomenon causes a decrease in the plasticizer amount in the polymer, with loss of elasticity and ductility of the material. Additionally, the leaking plasticizer from the material can contaminate the surrounding medium (medical application problems). Several factors influence the migration of the plasticizer, the main ones with regard to the type and concentration of the plasticizer, its molecular weight, branching, and polarity. Low-molecular-weight plasticizers are more prone to migrate from the polymeric material. The more linear the plasticizer structure, the faster the extraction and migration rate will be compared to more branched plasticizers. The migration of the plasticizer is also influenced by the type of polymer, its molecular weight, and its compatibility with the plasticizer, and from the plasticization process and the homogeneity of the product.

### PHB Physical Blends and Reactive Blends

For a sustainable processing strategy, the blending with other polymers or additives represents a much more easy and cost-effective approach and, as a result, is the more frequently used technique in the industrial sector (Anna and Arrigo, 2019). In fact, following this procedure, polymeric materials with enhanced and tailored chemicophysical and mechanical properties can be obtained by opportunely regulating the weight ratio between the selected polymers. Actually, the blending of two polymeric matrices is strictly correlated to their miscibility, i.e., to their respective solubility parameters (δ). If they are quite similar, a good miscibility should be expected. Anyway, the miscibility between two or more polymers also depends on the processing temperature, weight ratio in the blend compositions, as well as polymer respective molecular weights and crystallinity (Arrieta et al., 2017). Among bio-based polymers, poly(lactic acid) (PLA) is the most used mainly in food packaging area because of its easy processability, high transparency, and relatively low costs. PLA is produced through fermentation of lactic acid, followed by chemical polymerization; differently from PHB, it is a compostable material mainly commercialized for single-use disposal packaging items, such as bottles, cold food cups, and trays, as well as for flexible films (Auras et al., 2004; Jamshidian et al., 2010). Blending PHB with PLA could gain mutual advantages; indeed, PLA shows low crystallinity, scarce barrier properties, low heat distortion temperature (softening above 60°C), and difficult biodegradation at ambient conditions. These drawbacks, limiting its industrial exploitation, could be improved by its melt blending with another highly crystalline biopolymer matrix with similar melting temperature, lower Tg, and suitable barrier properties and biodegradability, as in PHB (González-Ausejo et al., 2017). Hence, PHB/PLA blends have gained a great interest, since their combination allows to obtain new biopolymer based systems with enhanced properties as compared to the single components while preserving their ecosustainability (Modi et al., 2013). Nevertheless, generally, PHB and PLA do not evidence fine miscibility although they have similar solubility parameters, ranging between 19.5 and 20.5 (MPa^1/2^) for PLA and between 18.5 and 20.1 (MPa^1/2^) for PHB. Actually, in polymer blending, several parameters should be taken in consideration. The first one is the polymer molecular weight. In their paper, Blümm and Owen found that low-molecular-weight (LMW) PLA and high-molecular-weight (HMW) PHB were melting miscible over the whole composition range, whereas a blend of HMW-PLA with LMW-PHB evidenced phase separation above PHB content of 25% (Blümm and Owen, 1995).

Similarly, Zhang and Thomas (2011) reported the formulation of PLA/PHB blends with different weight ratios (100:0, 80:20, 60:40, 40:60, and 0:100), showing that some interactions between the two polymers were established, notwithstanding their immiscibility. In particular, the blend containing 25 wt% of PHB evidenced outstandingly improved tensile properties compared with pure PLA because of the homogeneous dispersion of PHB crystals, which acted as a filler and nucleating agent in PLA. Anyway, the processing temperature can play a decisive role on PHB-PLA miscibility by providing, in some case, to melt reactive compatibilized blends. Indeed, while LMW-PLA and HMW-PHB blends obtained by solvent casting at room temperatures evidence phase separation in all compositions, the same blend melt blended at 200°C showed higher miscibility, evidenced by the lowering of both PHB melting temperature and PLA glass transition temperature The better miscibility was ascribed to transesterification reactions occurring between PLA and PHB chains during heating, leading to the formation *in situ* of low-molecular-weight PLA-PHB block copolymers acting in the interfacial region between the two phases as enhancer of their compatibility (Zhang et al., 2012). In addition, PHB/PLA interfacial compatibilization can be improved by melt-reactive extrusion in the presence of an external compatibilizer agent. For instance, Jandas et al. (2014) used maleic anhydride (MA) as reactive compatibilizer for PLA and PHB matrices to increase their miscibility. Actually, MA chemically grafted the α-carbon atom of the carbonyl group of PHB and PLA. Moreover, the presence of the reactive dicumyl peroxide was responsible of the formation of cross-linked and branched structures at their interfaces. In addition, the compatibilized blends evidenced substantial improvement of mechanical flexibility as a function of MA concentration. Passing from 1 to 9 wt%, PLA-PHB 70:30 blend changed from a brittle to a ductile material, reaching the best flexibility of more than 500% by grafting 7 wt% of MA (Jandas et al., 2014).

In order to improve PHB-PLA blends flexibility, by both increasing the polymer chain mobility and improving their processing for film manufacturing, plasticizers are frequently added to the blend. In this sense, PLA-PHB plasticization has been proven to be an effective way to enhance mechanical performance as well as to improve the compatibility between PLA and PHB biopolymers. Nowadays, the traditional plasticizers give way to natural ones due to the migration phenomenon, which could result in potential human health and environmental hazards (Harmon and Otter, 2018). Several plasticizers have been used mainly at concentrations between 10 and 30 wt% for film applications, such as glycerol (Martin and Avérous, 2001), poly(adipates) (Martino et al., 2011), PEG (Wang et al., 2008), citrate esters (Fenollar et al., 2013), and low-molecular-weight additives such as aroma compounds including D-limonene, carvacrol, and thymol (Arrieta et al., 2014). Most of them were able to decrease PLA Tg and increase polymer blend tensile strain (Arrieta et al., 2017), as previously detailed.

The blending with another natural polymers, such as thermoplastic starch (TPS), could represent a valid support to both obtain cost-effective biopolymer-based systems and enhance PHB properties without compromising environmental and carbon management benefits. Starch, a biodegradable polysaccharide produced by numerous plants, is one of the most abundant renewable feedstock resources known to man. Thanks to its biodegradability, renewability, and easy availability, starch has been extensively studied as a low-cost component of biodegradable plastic materials (Zhang et al., 2014). It is mostly composed of linear amylose and highly branched amylopectin organized in granular state due to the inherent hydrogen bonding between molecules. This native structure, providing a high crystalline material, severely hinders the dispersion of starch into a polymer matrix at a fine scale. When mixed with some water and/or plasticizers such as glycerol and following subjection to heat and shearing action, starch undergoes spontaneous destructurization, provoking the breaking down of intermolecular hydrogen bonds in favor of the polymer gelatinization. A homogeneous melt known as thermoplastic starch (TPS), evidencing typical thermoplastic behavior, is thus formed (Pyshpadass et al., 2008). Hence, TPS can be obtained and formulated by means of standard equipment commonly used in industrial manufacturing of synthetic polymers (Liminana et al., 2019). However, the high hydrophilicity and hygroscopicity, the quick physical aging effect due to the retrogradation process, and the poor mechanical properties strongly affect the industrial application of neat TPS (Ortega-Toro et al., 2017). This is why blending with more hydrophobic polymers, such as PHB, could enhance its functional properties (Turco et al., 2019a).

Hence, the mixing of PHB and TPS would combine the advantages of the two polymers by synergizing their properties while preserving the complete biodegradability of the blend. In their work, Godbole et al. (2003) blended PHB and TPS at different weight ratio compositions and studied the thermal and mechanical properties of the obtained films. They found that the blends PHB/TPS with a w/w percentage ratio of 70:30 showed a consistent improvement of mechanical performances with respect to neat PHB; as an example, the blending of only 30% w/w of TPS to PHB could double and quadruple the stress and strain at break values of the polyester, respectively. Although no shifting of PHB melting temperature could be observed, thus indicating that there were no interactions at molecular level, a strong delay of PHB decomposition temperature could be highlighted passing from about 220 to 260°C; TPS could act as a thermal stabilizer of PHB.

Vice versa, by investigating the influence of PHB on TPS properties, Lai et al. (2006) studied the mechanical properties of films based on TPS doped with different amounts of PHB (1, 3, 5, and 7 wt%). They found that the mechanical properties of films increased by increasing PHB content, whereas the values of water absorption, one of the main issues of hygroscopic TPS, decreased because of the higher PHB hydrophobicity. Actually, it is worthy to underline that, for PHB/TPS blends, there is an optimized ratio between glycerol (used to thermoplasticize starch) and PHB concentration finalized to reach a suitable compatibility between the polymers. The same authors found that, when TPS contains 25% of glycerol, the tensile strength of the blend TPS/PHB significantly increased by increasing PHB content, whereas when 33% of glycerol is used, only slight changing could be observed. This outcome could be probably due to the higher gelatinization degree, resulting in a structure more prone to water diffusion. Similar results were found by Thiré et al. (2006). In their work, the authors investigated compression-molded PHB/starch blends with starch content varying between 0 and 50%. They found that, above 30% in weight of TPS blended with PHB, higher starch contents led to the worsening of mechanical properties due to the lack of interfacial adhesion between starch and PHB, also evidenced by morphological analysis.

In order to improve the interfacial adhesion between the polymers, Zhang and Thomas (2010) prepared PHB/starch blends by melt process and investigated the properties in terms of interfacial adhesion between the polymers. Two types of maize starch, starch 1 (containing 70% amylose) and starch 2 (containing 72% amylopectin), were melt blended with PHB. The spectroscopic and morphological analyses evidenced that starch particles acted as nucleating agent for PHB crystallite formation by significantly reducing the spherulites size; moreover, the starch fillers physically interacted with PHB by means of intermolecular hydrogen bonds. This effect was particularly highlighted when the more linear and tighter structure of amylose-rich starch was used. Hydrogen bonding between the polar residues of starch (hydroxyl groups) and carbonyl residues of PHB could inhibit the chain scission degradation in PHB, thus improving its thermal stability. As a consequence, higher melt shear viscosity and better mechanical properties were observed when starch 1 was used.

### PHB Reactive Blends

Blending different polymers opens up a range of possibility for the development of novel materials with interesting properties. As previously underscored, melt compounding is one of the most effective methods to tune the polymer blending properties. This method allows preparation of new bio-based polymer blends or composites with pleasing properties. Anyway, most of the blends consist of thermodynamically immiscible polymers, and the simple physical mixing in the melt state usually leads to phase separation on the micrometer scale resulting in unfulfilling properties of the final products. To overcome the problems related to incompatibility, bio-based polymer blends and composites require a reduction in interfacial tension between the components leading to improved final properties of the materials (Muthuraj et al., 2018). Among various strategies for compatibilization, reactive blending seems to be one of the most promising and environment-friendly approaches (Raquez et al., 2008). It is a fast, solvent-free, low-cost, and environment-friendly method by which designed chemical reactions between the components in suitable processing conditions occur. Reactive blending is really versatile since it can be successfully applied during “*in situ* polymerization” of biodegradable polymers, functionalization of natural fibers/fillers, or chemical modification of the polymer structure. Moreover, the reactive blending occurring by using the “dynamic curing” of bio-based polymers and composites is worthy of consideration in this research field. The method consists on using reactive species as organic peroxides in the melt chamber resulting in the formulation of copolymers, which act as interface compatibilizers between two (or more) polymer matrices. In the literature data, the dynamic curing of biodegradable aliphatic polyesters blends (Ma et al., 2014), aliphatic polyester/natural rubber blends (Wang et al., 2015), and, more recently, bio-composites (Luo et al., 2016) indicated that the high temperature necessary to decompose organic peroxides also induces transesterification reactions of aliphatic polyesters, responsible of polymer chain rearrangements toward soluble copolymers. This confirms that dynamic curing of bio-based polymer blends and composites can lead to the development of semi-interpenetrating networks connecting two (or more) phases, which significantly enhances their physicomechanical properties.

In their paper, Przybysz et al. (2018) used two different organic peroxides, dicumyl peroxide (DCP) and di-(2-tert-butylperoxyisopropyl)-benzene (BIB), to compatibilize polycaprolactone (PCL)/PHB blend. Polycaprolactone is a synthetic semi-crystalline linear aliphatic polyester that is biocompatible and biodegradable. It is ductile and has a significantly lower melting point than PHB at about 60°C. Blends of PCL and PHB have attracted much attention due to their inherent biodegradability and biocompatibility, although they are immiscible on the molecular scale as proven by Antunes and Felisberti (2005).

It was observed that the addition of free radical initiators to PCL/PHB blends resulted in the significant enhancement of mechanical and thermal properties in comparison to uncompatibilized blend obtained by only physical mixing. The severe improvement of PCL/PHB properties was due to the partial cross-linking and/or branching of the covalently linked blends, as evidenced by the melt flowrate. Moreover, the better efficiency of BIB due to its higher number of free radicals was responsible for a tightened final compatibilized structure (Przybysz et al., 2018).

### PHB-Based Bionanocomposites

One of the main drawbacks of PHB is the high costs correlated to the fermentation and extraction processes. As a result, several studies investigated on the preparation of PHB bio-composites by blending it with natural fibers and fillers, aiming at producing more cost-effective materials with improved properties (Delmas et al., 2011; Garcia-Garcia et al., 2016). Inspired by these considerations and in the frame of an eco-sustainable approach, Angelini et al. (2014) prepared compression-molded bio-composites employing a lignin-containing filler obtained as a by-product of the bioethanol fermentative production process, using *Arundo donax* as a biomass (Vishtal and Kraslawski, 2011). When blended with biodegradable aromatic and aliphatic polyesters, an increase in thermal stability and elastic moduli of the resulting composites was observed, not neglecting the valid opportunity for converting an agro-food by-product into a bio-resource, aimed to improve the properties of PHB in an environment-friendly and cost-effective way (Mousavioun et al., 2010). Therefore, in order to reduce the costs of filler processing, hence the bio-composite overall cost, no pretreatments other than milling were performed on the crude lignin-containing residue filler prior to use. The biodegradation behavior of the composites, qualitatively assessed by analyzing the surface of soil buried films, evidenced a significant surface degradation of PHB-based bio-composites. The authors demonstrated that lignin positively affected the rheological behavior of the polymer melt and acted as a PHB nucleating agent (Angelini et al., 2014). In mixtures, the nucleation rate and size of the spherulite depend on the cooling rate and the nucleation density. Nucleating agents (NAs) provide polymers with homogeneous nuclei and allow more rapid crystallization during the cool-down period after the polymer melts. Indeed, it is very important to both accelerate the crystallization rate of PHB-based materials and provide the formation of small and more homogeneous spherulite sizes in order to enhance its mechanical properties In general, adding NAs results in an increase in melt crystallization temperature and narrowing of the crystallization peak during non-isothermal melt crystallization; in other words, in the presence of NA, less degree of supercooling and shorter crystallization time occur (Shi et al., 2015).

Recently, natural fibers are increasingly being utilized as environment-friendly materials to improve polymer properties. The natural fibers can represent a valid alternative to synthetic oil–fossil-derived fibers, such as glass and carbon fibers, commonly used as reinforcements in bio-plastics due to their strong mechanical properties. The cellulose-based fibers are eco-sustainable and cost effective (Tan et al., 2015) due to their biodegradability, renewability, and availability; moreover, they have low density, competitive specific mechanical properties, and a relatively low cost (Ching et al., 2016). Generally, along with a number of benefits as reinforcements, there are some drawbacks associated with the exploitation of these natural lignocellulosic fibers. Indeed, the hydrophilicity and strong cross-linking of lignocellulosic fibers prevent the compatibility with biopolymer matrices, leading to poor interfacial adhesion and mechanical properties (Sanchez-Garcia et al., 2008). In their paper, Tănase et al. (2015), prepared new bio-composite materials based on PHB and different percentages of cellulose fibers (from 2 to 10%) in order to improve PHB physical and mechanical behavior. By increasing the cellulose fiber content, the authors evidenced a decrease in melt viscosity and melting temperature, making the composites easy to process.

The crystallinity of PHB/FC composites decreased for all samples compared to neat PHB. The decrease in the crystallinity of the tested samples could be attributed to the hindered motion of the polymer segments due to the presence of cellulose fibers in the polymer matrix. The composite showed a blocking effect in the UV light spectra region while maintaining high transparency, resulting in a suitable property for a packaging material. On the other side, the water vapor barrier was poor due to the hydrophilicity of cellulose fibers (Tănase et al., 2015).

Driven by the necessity to overcome these issues, attempts to improve bio-composite performances have been carried out by the development of bionanocomposites. Polymer nanocomposites, obtained by incorporation of nanosized particles into polymer matrices, evidenced clear improvements of PHB properties, mostly if mechanical performances are concerned. For example, studies on the effect of organoclay minerals on mechanical properties of PHAs have been performed (Ozkoc and Kemaloglu, 2009). As a matter of fact, polymer nanocomposites exhibited markedly improved properties when compared to the pure polymer. Mineral clays are the most used silicates in bionanocomposites manufacturing. Indeed, they can modify the polymer characteristics and improve their processability. As clays are naturally hydrophilic, in order to make them more compatible with hydrophobic polymer like PHB, the cations between the layers can be changed by cationic surfactants like the alkyl ammonium (Alexandre and Dubois, 2000). The modified clay becomes organophilic, its surface energy decreases, and the interbasal distance increases. The modified organo-nanosilicates are more compatible with the hydrophobic PHB nature due to the improvement of the interfacial adhesion. Furthermore, it makes possible the polymer molecules intercalating inside clay galleries, thus strongly modifying the mechanical, thermal, and barrier properties of the polymer (Bordes et al., 2009a).

Among the traditional composites, bentonite is one of the lamellar silicates most used as inorganic filler. Indeed, it is environment friendly and available in large quantities at a relatively low cost. Thus, it provides for the manufacture of bionanocomposites, preserving the biodegradability of the whole system (Bordes et al., 2009b). In their paper, Júnior et al. evaluated the thermal behavior of PHB/PEG/clay bionanocomposites. It was observed that the initial temperature of degradation of bionanocomposites increased with organobentonite content. It was also verified that clay addition to most of the systems led to an increase in crystallinity compared to the PHB matrix, which was attributed to clay nucleating action able to reduce the size of the spherulitic crystals by both decreasing the free energy barrier required for crystal growth and increasing the number of nucleation sites available for crystal growth. This effect was due to the organic modification of the bentonite able to provide suitable different interlayer structures for the bionanocomposites (Júnior et al., 2019). Actually, from the application point of view, PHBV is widely investigated in the preparation of high-performing nanocomposites to be exploited in food packaging. However, the main drawbacks needed to be overcome are its slow crystallization rate and low crystallinity responsible for the dropping down of mechanical and barrier properties. According to Zhang et al. (2017), the addition of cellulose nanocrystals (CNCs) substantially modulates the crystallization profile of the polymer, thus enhancing its mechanical property and thermal stability. In addition, finalized to bestow antibacterial properties, thus obtaining a bioactive food packaging material, the authors included bifunctional nanohybrids composed of CNCs and antibacterial agents, such as silver nanoparticles, AgNPs, in the biopolymeric matrix and found that they acted as suitable nucleating agent able to strongly improve PHBV crystallization temperature and rate and whole crystallinity. As a consequence, also thermal stability, mechanical, barrier, and antibacterial properties of the ternary bionanocomposite significantly enhanced.

In their paper, Yu et al. (2010) performed a pioneering research about the structural and optical properties of PHBV/ZnO nanofibers fabricated by the electrospinning technique. ZnO nanoparticles (NPs), doped in the PHBV fibers, resulted in well-dispersion due to the hydrogen bonding occurring between the polar groups of ZnO and PHBV. The crystallinity and crystallization rate were lowered by adding ZnONPs.

Based on the previous research, recently, Castro-Mayorga et al. (2017b) investigated the effect of zinc oxide size, morphology, and crystalline structure on their antimicrobial activity against the foodborne pathogens *Salmonella enterica* and *Listeria monocytogenes* of PHBV for active food packaging and food contact applications. They found that ZnO particles were significantly effective when their specific surface area increased, i.e., when the hexagonal-pyramid nanoparticles (PZnO) were used. In addition, they evidence that the antibacterial properties were preserved even when they were incorporated in coating PHBV structures. Moreover, ZnO nanoparticles positively influenced the thermal stability and optical properties of PHBV active films, avoiding their browning after the thermal processing, even if they worsened the polymer mechanical and oxygen barrier properties due to the high concentration required to obtain suitable bactericide effect against *L. monocytogenes*.

In order to improve PHBV gas barrier properties, which is useful for food and electronic packaging materials, and its sensitivity to oxygen, many efforts have been performed. The introduction of nanosized fillers inside polymeric matrix attracted a great interest since their fine and homogeneous distribution inside the polymer hinders gas permeability. In their paper, Öner et al. (2016) used boron nitride crystals in hexagonal nanoplate and nanoflake due to their structural similarities to graphene. BN nanoparticles included in the PHBV matrix during the extrusion process improved the gas barrier of the polymer. Anyway, in order to enhance the physical interaction occurring between PHBV and BNPs, thus assuring a stable dispersion of BNPs, the same authors used a silane coupling agent, octyltriethoxysilane (OTES), to modify the surface of boron nitride. In their research, Öner et al. (2019) included different amounts of BNPs with different crystalline silane surfaces in PHBV, via melt compounding, and investigated their effects on the barrier properties of PHBV nanocomposites. For all the nanocomposites, the oxygen permeability decreased in comparison to the neat PHBV due to the presence of both BN nanoparticles and silane coupling agent. In particular, the physical interaction occurring between PHBV and BN were strongly enhanced in the presence of OTES, furthermore improving the barrier properties.

Similarly, Öner and Ilhan (2016) investigated the chemical–physical properties of bio-composites based on PHBV and hydroxyapatite (HAP), obtained by extrusion processing. HAP is one of the bioactive and biocompatible calcium phosphates fillers widely used for polymer nanocomposites. Since both synthetic and natural HAPs have the same chemical composition and crystallographic properties of bone joint, it is widely used as osteoconductive filler for bone regeneration (Basile et al., 2015).

The bio-composites, produced by melt extrusion of PHBV with untreated HAP and silane surface-treated HAP crystals, were investigated by structural, morphological, and thermal analyses. At high loading of unmodified HAP nanoparticles, physical agglomeration occurred, and mechanical properties dropped down, as the lack of proper adhesion between the matrix and the HAP results in insufficient stress transfer. The silane coupling agent induced a better dispersion of HAP NPs inside PHBV, as evidenced by SEM analysis; as a consequence, a good stress transfer from the matrix to the dispersed phase could be observed. Thus, surface treatment by silanization proved to be necessary to avoid a decrease in the mechanical properties of the filled biopolymer matrix (Öner and Ilhan, 2016).

Finally, Ambrosio-Martín et al. (2015a,b) prepared novel nanocomposites based on PHBV and functionalized graphene nanosheets (FGS) by means of mechano-physic ball milling method. Morphological characterization evidenced proper nanofiller dispersion into the matrix, whereas DSC analysis evidenced an increased crystallinity of the polymer. Thermal stability tests revealed that FGS affected the mechanism of oxidative thermal degradation while they did not influence the thermal degradation by pyrolysis. The authors highlighted the positive influence of the homogeneous dispersion of FGS nanofiller within the polymeric matrix, for both the mechanical reinforcing effect of FGS and also gas barrier enhancement (Ambrosio-Martín et al., 2015a,b).

## Challenges and Perspectives for Effective PHA Exploitation

The main challenge for exploitation of PHA polymers is related to their high production costs. The costs of the raw materials and recovery process are ~10 times higher than those of conventional polymers, with the carbon sources used to feed the fermentation process accounting for more than the 50% of the total (Wang et al., 2013a; Koller et al., 2017). An estimation of the biopolymer costs is ~$6–15/kg, almost two orders of magnitude higher than those of PE and PP ($0.23–0.48/kg) (Reddy et al., 2003).

An important aspect related to the cost competitiveness of PHA production is the final process productivity, which in turn depends on production yield as well as on the efficiency of downstream procedure. Genome-editing and synthetic biology approaches have emerged as powerful and innovative tools to address both these aspects (Zhang et al., 2020). Ribosome-binding site (RBS) optimization, promoter engineering, and, more importantly, CRISPR-cas9-based approaches have been effective in model organisms, such as *E. coli* and non-model ones, such as *Halomonas* spp. and *Pseudomonas* spp., to boost process competitiveness (Zhang et al., 2020). Cell sizes and growth behavior are crucial targets for increasing polymer accumulation. Engineering cell size/shape or cell walls is an effective way to boost PHA accumulation within the intracellular space. CRISPR/cas9 and CRISPi methods have been used to manipulate genes related to cell division. The deletion of fission-related genes, together with overexpression of the genes involved in the division process in *E. coli*, allows to significantly increase PHB accumulation by inducing a multiple division pattern and, consequently, the formation of a cell with increased volume (Jiang et al., 2015; Elhadi et al., 2016; Wu et al., 2016a,b). Cell shape is also an important factor influencing further cell separation from the culture broth. A convenient downstream process has been designed in engineered *Halomonas campaniensis* LS21,by deleting an *etf* operon encoding two subunits of an electron transferring flavoprotein, causing self-flocculation and an easy and rapid recovery at the bottom of the bioreactor (Ling et al., 2019; Shen et al., 2019).

Biomass feedstocks and waste materials have emerged as promising substrates for cost-effective PHA production, and their different kinds have been explored, including lignocellulosic materials and agroindustrial and food wastes (El-malek et al., 2020; Sirohi et al., 2020). Progresses in this field have been achieved through the application of *in vivo* engineering approaches. Microbial cell factories able to convert different wastes into PHA have been designed, focusing on different approaches: (i) introducing the catabolic genes required for the metabolization of waste C-sources in native PHA producers; (ii) conferring PHA-producing abilities to non-native producers endowed with advantageous metabolic/physiological features, i.e., halophilic bacteria and/or microorganisms naturally able to metabolize complex C-sources; (iii) modulating PHA composition acting on precursor-supplying pathways (Favaro et al., 2019).

Although PHA at industrial scale is currently produced by pure culture systems (wild-type or engineered microorganisms), the use of MMC is emerging as a promising strategy to reduce the intensive costs related to aeration and media and equipment sterilization (Fradinho et al., 2019; Mannina et al., 2020). A further advantage of MMC-based processes is the possibility to integrate PHA production in waste treatment plants (Kourmentza et al., 2017). In one of the most promising examples, up to 75 wt% of PHA content has been reported by MMC from fermented molasses (Albuquerque et al., 2010). It has been estimated that, by optimizing the acidogenic fermentation to get a large amount of organic acids from organic fraction of municipal solid waste (OFMSW) to be further transformed into PHA, a total gross revenue of $7.6–16.9 billion might be achieved (Colombo et al., 2017). The feasibility of the MMC-based processes on waste substrates has been demonstrated at pilot scale, and several European projects aim at demonstrating PHA production at pilot scale have been funded (Mannina et al., 2020). Future improvements for the exploitation of MMC-based processes should address the increase in cell densities and polymer productivities (Argiz et al., 2020) as well as the improvements in the extraction technologies due to their higher resistance to cell hydrolysis with respect to pure cultures (Samorì et al., 2015; Mannina et al., 2020).

Most of the reported studies focus on the successful design and optimization of the processes for PHA production from wastes on lab-scale; however, an estimation of process costs has been reported only in few cases. In an interesting example, PHB process economics using cheese whey as the low-cost substrate was simulated on scenarios with different plant capacity. The bioprocess was found efficient at 1,000 ton/h whey feed with an estimated cost of US$10.2/kg (Peña-Jurado et al., 2019).

Although the use of waste materials certainly allows to reduce the costs of raw material supply, the PHA production process is still not economically competitive. Exploiting microbial cell factories to obtain multiple products from the same process would enhance process competitiveness. Several examples of coproduction of PHA with other value-added products (amino acids, proteins, alcohols, hydrogen, biosurfactants, exopolysaccharides, and lipids) have been described in the recent literature (Kumar and Kim, 2018; de Jesus Assis et al., 2019). From an economical and technical point of view, the most advantageous processes are those that couple an optimized accumulation of intracellular PHA together with the recovery of extracellular products (Kumar and Kim, 2018). A further breakthrough in the same direction is represented by the implementation of “waste bio-refineries,” wherein the PHA production is integrated in an ensemble of processes aimed at the complete valorization of raw materials. A techno-economic and environmental analysis of a sugar cane bagasse biorefinery for the production of fuel ethanol, PHB, and electricity was performed by Moncada et al. (2013). An optimization procedure was applied to select the most promising process pathways for each product and to define the criteria for the selection of technologies and raw material distribution. It was demonstrated that, if considered in the frame of a multiproduct biorefinery, where all the products contribute in incomes and also share costs, instead of a separate process, the PHA production becomes a feasible process.

A similar approach was applied to evaluate the economic viability of a biorefinery for the coproduction of succinic acid, PHB, and electricity from sugarcane bagasse and trash lignocelluloses (Nieder-Heitmann et al., 2019). Different scenarios were simulated, and the most favorable configuration (where PHB was produced from 25% of the fermentable glucose stream and succinic acid from the remaining glucose together plus the hemicellulose hydrolysate) resulted in an internal rate of return (IRR) of 24.1% with a net present value of US$477.2 million.

Although the above-described strategies of PHB production from renewable, eco-sustainable, and cost-effective bioresources are ever more biotechnologically advanced and promising approaches to drastically reduce the high production cost of PHB, its commercialization is still in its early stages, even if its global production capacity is one of the fastest growing among biopolymers (Aeschelmann and Carus, 2017). This is due to the restricted areas of PHB application unavoidably associated to the lost challenge in up-front competition with the petroleum-based plastics produced on a very large scale. A valid attempt to obtain a cost-competitive material is to strongly broaden its industrial production and diffusion in bioplastics industry. A lot of progress has been recently made through the formulation of PHB with tailored additives (plasticizers, nucleating agents, organic, and inorganic fillers), polymers (physical blending and/or melt reactive blending), copolymers, and bio-composites, leading to greatly improved mechanical profiles, wider processability windows, and improved post-processed thermal stability, without neglecting that PHB compounding requires lower amounts of the neat polymer with a consequent reduction in the final material costs. These advances will improve PHB capacity to match with the practical needs in several application fields, ranging from surgical sutures, tissue engineering (Misra et al., 2010), agricultural foils, and packaging materials for the storage of food products (Bucci et al., 2005).

In this way, PHB could both shed lights on its commercial applications and enhance its penetration in wider market sectors, with a consequent reduction of its high production cost.

Nevertheless, it is expected that the PHA production will almost quadruple by 2021 with regard to 2016, as a result of a ramp-up of capacities in Asia and the USA (www.euroepanbioplastics.org/market), and thus, it seems that the costs of PHAs will decrease. This upcoming perspective is the result of an important trend, driven by the changing of consumer demands to make plastic products more efficient, eco-friendly, and cost effective and to reduce greenhouse gas emissions and dependency on fossil-derived plastics.

## Author Contributions

RT: conceptualization, writing (review and editing), and special focus on plasticizers. GS: conceptualization, writing (review and editing), special focus on blending, and bio-composites. IC: investigation and visualization. CP: conceptualization, writing (review and editing), special focus on copolymer synthesis and *in vivo* approaches, and supervision. MD: funding acquisition. All authors contributed to the article and approved the submitted version.

## Conflict of Interest

The authors declare that the research was conducted in the absence of any commercial or financial relationships that could be construed as a potential conflict of interest.

## Abbreviations

3H9D: 3 hydroxy-9-decenoate
9DEO: 9-decenol
ATBC: acetyl tributyl citrate
AVF: aloe vera fibers
BDO: 1,4 butanediol
BIB: di-(2-tert-butylperoxyisopropyl)-benzene
CT: chain transfer
DBS: dibutyl sebacate
DCP: dicumyl peroxide
DDA: dodecanoic acid
DHBA: 2,3-dyhydroxybutyrate
DOS: dioctyl sebacate
EB: elongation at break
FC: cellulose fibers
FDA: federation
G: geraniol
GA: geraniol acetate
HB: hydroxybutyrate
HD: hydroxydecanoate
HDD: hydroxydodecanoate
HHx: hydroxyexanoate
HP: hydroxyproprionate
HPhV: hydroxy-3-phenylvalerate
HTD: hydroxytetradecanoate
HV: hydroxyvalerate
IRR: internal rate of return
L: linalool
L503: Lapro1503
LA: levulinc acid
MA: maleic anhydride
NA: nucleating agents
NCS: neural stem cells
PCL: polycaprolactone
PCT: poprionyl CoA transferase
PDLLA: poly(DL-lactide)
PDO: 1,3 propanediol
PE: polyethylene
PEG: polyethylene glycol
PhaP: PHA binding protein
PLA: polylactic acid
PP: Polypropylene
PVA: 5-phenylvaleric acid
RBS: ribosome-binding site
RGD: arginyl-glycyl-aspartic acid petide
TBC: tributyl citrate
Tc: crystallization temperature
TC: triethyl citrate
Tcc: cold crystallization temperature
Tg: glass transition temperature
Tm: melting temperature
YM: Young's modulus
δ: solubility parameter.
